# Supplementary material for: Genetic studies of accelerometer-based sleep measures yield new insights into human sleep behaviour
Source: Nat Commun. 2019 Apr 5;10:1585. doi: 10.1038/s41467-019-09576-1 (PMC6451011; doi:10.1038/s41467-019-09576-1)
Supplement: Supplementary file 1 — Supplementary Information [file 41467_2019_9576_MOESM1_ESM.pdf]

Genetic studies of accelerometer-based sleep measures yield new insights into human sleep behaviour

Jones, van Hees, Mazzotti et al

Supplementary Methods

Sensitivity analysis details

<u>*Lifestyle factors for sensitivity analysis (6)*</u>

In the sensitivity analysis that included “lifestyle” factors, we adjusted models for multiple variables measured at baseline that may have had an impact on sleep timing, quality and/or quantity. These were:

- Townsend Deprivation Index (TDI; field 189)
- Alcohol intake frequency (field 1558; categories treated as ordinal scale – “Never”=0 to “Daily or almost daily”=5)
- Smoking status (field 20116; categories treated on a nominal scale)
- Snoring (field 1210; nominal scale)
- Nap during day (field 1190; nominal scale)
- Had menopause (field 2724; nominal scale; included both “Not sure” answers as separate categories; males were recoded as an additional category to avoid exclusion)
- Caffeine intake (continuous scale; calculated from fields 1498, “Coffee intake”, and 1488, “Tea intake” using the formula caffeine_intake=137*coffee_intake + 47*tea_intake; individuals were assumed to have zero caffeine contribution from coffee if they answered “Decaffeinated coffee (any type)”, “Do not know” or “Prefer not to answer” in field 1508, “Coffee type”)
- Sleep apnoea (matching code 1123 in “Non-cancer illness code, self-reported”, field 20002; treated as binary variable)

Individuals responding “Do not know” or “Prefer not to answer” for “Alcohol intake frequency”, “Smoking status”, “Snoring”, “Nap during day” and “Had menopause” were excluded from this sensitivity analysis. Caffeine intake was representative of habitual intake and did not consider dietary recall questions, which were available in less than half of the cohort at the time of analysis.

<u>*Exclusion criteria for sensitivity analysis (7)*</u>

Shift or night shift work

Two baseline questionnaire variables were used to assess whether individuals were performing either shift work or night shift work at the time of assessment. Individuals answering anything other than “Never/rarely” for either field 826 (“Job involves shift work”) or field 3426 (“Job involves night shift work”) were considered to be shift or night shift workers.

Self-report mental health and sleep disorders

We excluded participants if they had answered yes in field 2090 (“Seen doctor (GP) for nerves, anxiety, tension or depression”) or had reported “sleep apnoea” (coded as 1123), “insomnia” (1616), “depression” (1286), “schizophrenia” (1289) or “mania/bipolar disorder/manic depression” (1291) at the baseline verbal interview. These data are stored in field 20002 (“Non-cancer illness code, self-reported”).

Hospital episode statistics (HES) mental health and sleep disorder diagnoses

Individuals were excluded if they had one or more of the following diagnosis codes (or their respective sub-classifications) in either field 41202 (“Diagnoses – main ICD10”) or 41204 (“Diagnoses – secondary ICD10”):

- Major depressive disorder (MDD) – ICD10 codes F32 and F33

- Schizophrenia – F20 to F29
- Bipolar disorder – F30 and F31
- Anxiety disorders – F40 to F43
- Mood disorders – F30 to F39
- Sleep disorders – F51 (nonorganic) and G47

Relevant self-report medications

We excluded any individuals reporting one or more of the following medications (field 20003) at baseline. These include drug ingredients and brand names:

Sleep medications: oxazepam, meprobamate, medazepam, bromazepam, lorazepam, clobazam, chlormezanone, temazepam, nitrazepam, lormetazepam, diazepam, zopiclone, triclofos, methyprylone, prazepam, triazolam, ketazolam, dichloralphenazone, clomethiazole, zaleplon, butobarbital, diphenhydramine product, nytol, sonata

Antidepressants: amitriptyline, citalopram, fluoxetine, sertraline, venlafaxine, dosulepin, paroxetine, mirtazapine, escitalopram, trazodone, prozac, seroxat, cipralex, duloxetine, lofepramine, clomipramine, nortriptyline, imipramine, dothiepin, cipramil, amitriptyline, prothiaden, trimipramine, lustral, reboxetine, zispin, cymbalta, anafranil, doxepin, moclobemide, phenelzine, fluvoxamine, yentreve, triptafen, surmontil, tranylcypromine, allegron, edronax, molipaxin, mianserin, nardil, faverin, nefazodone, amitriptyline+chlordiazepoxide, isocarboxazid, manerix, maoi, sinequan, tranylcypromine+trifluoperazine, ludiomil, norval, tryptizol, fluphenazine hydrochloride+nortriptyline.

Antipsychotics: prochlorperazine, olanzapine, quetiapine, risperidone, chlorpromazine, trifluoperazine, amisulpride, sulpiride, seroquel, haloperidol, aripiprazole, stelazine, depixol, flupentixol, clozapine, promazine, risperdal, modecate, fluanxol, flupenthixol, zyprexa, zuclopenthixol, clopixol, largactil, abilify, fluphenazine, haldol, serenace, clozaril, cpz, perphenazine, levomepromazine, pericyazine, dolmatil, fentazin, fluphenazine, benperidol, pimozide, zaponex, denzapine, neulactil, thioridazine, dozic, fluspirilene, panadeine, sertindole.

Anxiolytics: zopiclone, diazepam, temazepam, zolpidem, nitrazepam, lorazepam, hydroxyzine, zimovane, phenergan, promethazine, buspirone, atarax, oxazepam, loprazolam, chlordiazepoxide, lormetazepam, ucerax, stilnoct, diazepam, buspar, alprazolam, librium, xanax, meprate, dalmane, clomethiazole, meprobamate, welldorm, amitriptyline+chlordiazepoxide, flurazepam, heminevrin, medazepam, neulactil, sinequan, almazine, atensine, carisoma, chloractil, chloral, dichloralphenazone, dormonoct, methyprylone, mogadon, rohypnol, tryptizol.

Supplementary Tables

Supplementary Table 1. Spearman's rank correlation statistics for activity monitor derived sleep traits and self-report hours slept and self-report chronotype (coded for increased morningness).

Trait	M10 timing	L5 timing	Sleep midpoint	Sleep duration	Sleep duration (SD)	Sleep efficiency	Diurnal inactivity	Number of sleep episodes	Self-report hours slept
**L5 timing**	0.460								
**Sleep midpoint**	0.383	0.591							
**Sleep duration**	0.012	-0.023	-0.039						
**Sleep duration (SD)**	0.059	-0.007	-0.102	-0.303					
**Sleep efficiency**	0.009	0.035	0.119	0.566	-0.165				
**Diurnal inactivity**	0.043	0.083	-0.105	0.269	0.098	-0.200			
**Number of sleep episodes**	0.036	-0.023	-0.054	0.142	-0.182	-0.250	0.202		
**Self-report hours slept**	0.025	0.010	0.008	0.189	-0.043	0.133	0.040	0.082	
**Self-report chronotype**	-0.277	-0.293	-0.237	0.027	-0.042	0.018	-0.044	-0.006	-0.005

Supplementary Table 2. Averages of the mean number of sleep episodes detected within individuals in the UK Biobank split by APOE Alzheimer's disease risk haplotypes and lower/upper age groups.

	All Subjects		Lower age group (<63.5 years)		Upper age group (=>63.5 years)	
**Haplotype**	**N**	**Average Mean Sleep Episodes (SD)**	**N**	**Average Mean Sleep Episodes (SD)**	**N**	**Average Mean Sleep Episodes (SD)**
APO-e2/e2	479	17.40 (3.70)	236	18.07 (3.59)	243	16.75 (3.69)
APO-e2/e3	8,922	17.32 (3.62)	4,386	17.64 (3.66)	4,536	17.00 (3.55)
APO-e2/e4	1,795	17.17 (3.59)	913	17.74 (3.67)	882	16.58 (3.40)
APO-e3/e3	42,679	17.26 (3.60)	21,210	17.63 (3.63)	21,469	16.90 (3.52)
APO-e3/e4	17,049	17.14 (3.60)	8,686	17.52 (3.65)	8,363	16.74 (3.49)
APO-e4/e4	1,660	17.02 (3.56)	862	17.49 (3.71)	798	16.52 (3.31)

Supplementary Table 3. MAGMA Gene-Set Analysis for SNPs associated with disturbed sleep based on number of sleep episodes reaching Bonferroni significance.

Gene Set	N genes in gene set	Gene set beta*	Gene set beta STD**	SE***	P-value	P_Bonferroni
GO_bp:go_serotonin_metabolic_process	10	1.43	0.0335	0.264	3.10E-08	3.4E-04
GO_bp:go_primary_amino_compound_metabolic_process	13	1.06	0.0283	0.232	2.34E-06	0.026

* Regression coefficient of the gene set
** Gene set beta STD: semi-standardized regression coefficient, corresponding to the predicted change in Z-value given a change of one standard deviation in the predictor gene set
*** Standard error of regression coefficient

Supplementary Figures

a)

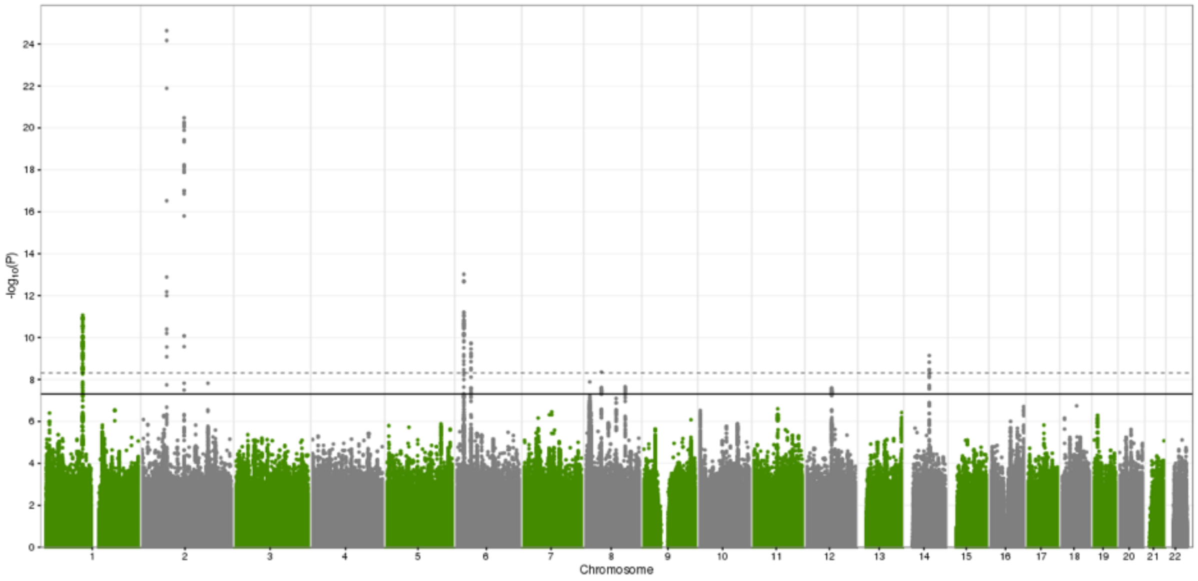

b)

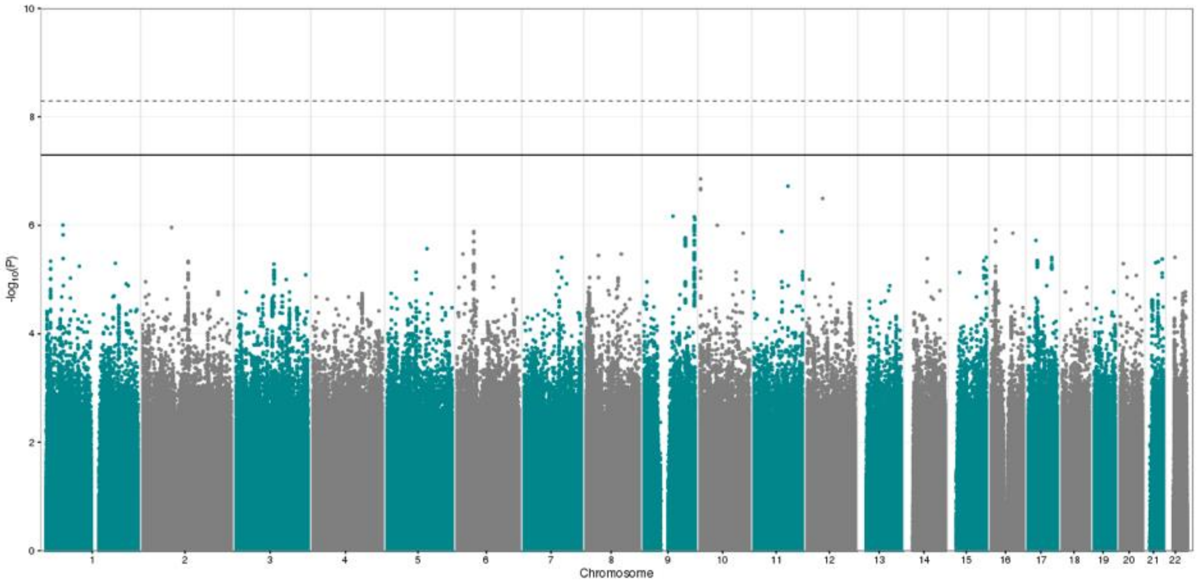

c)

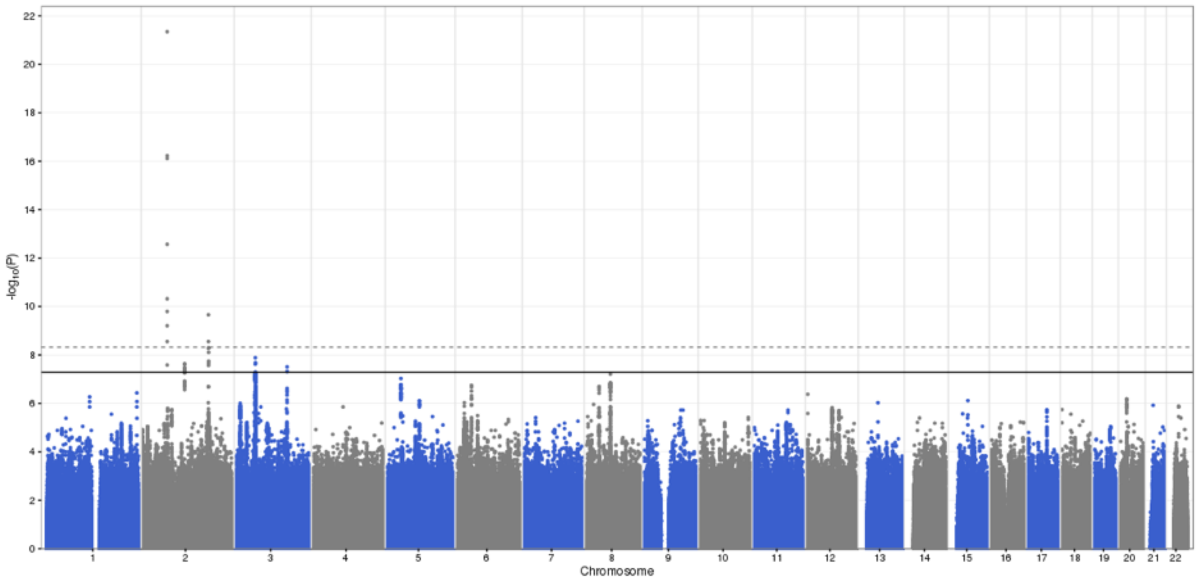

-log10(P)
Chromosome
1 2 3 4 5 6 7 8 9 10 11 12 13 14 15 16 17 18 19 20 21 22
0 2 4 6 8 10 12 14 16 18 20 22

d)

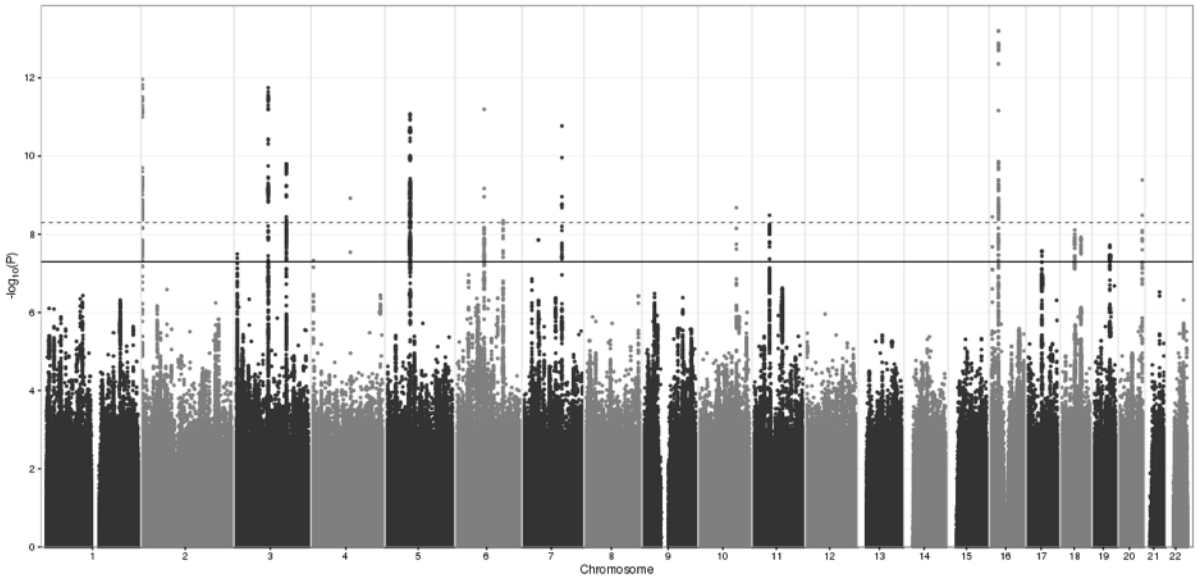

-log10(P)
Chromosome
1 2 3 4 5 6 7 8 9 10 11 12 13 14 15 16 17 18 19 20 21 22
0 2 4 6 8 10 12

e)

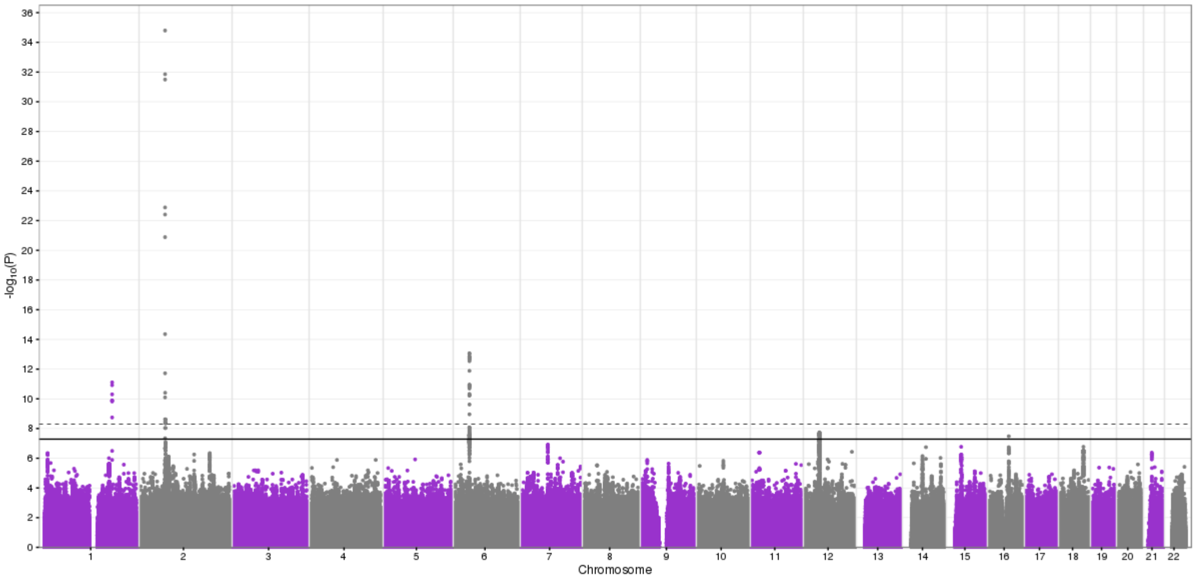
-log10(P)
Chromosome
1 2 3 4 5 6 7 8 9 10 11 12 13 14 15 16 17 18 19 20 21 22

f)

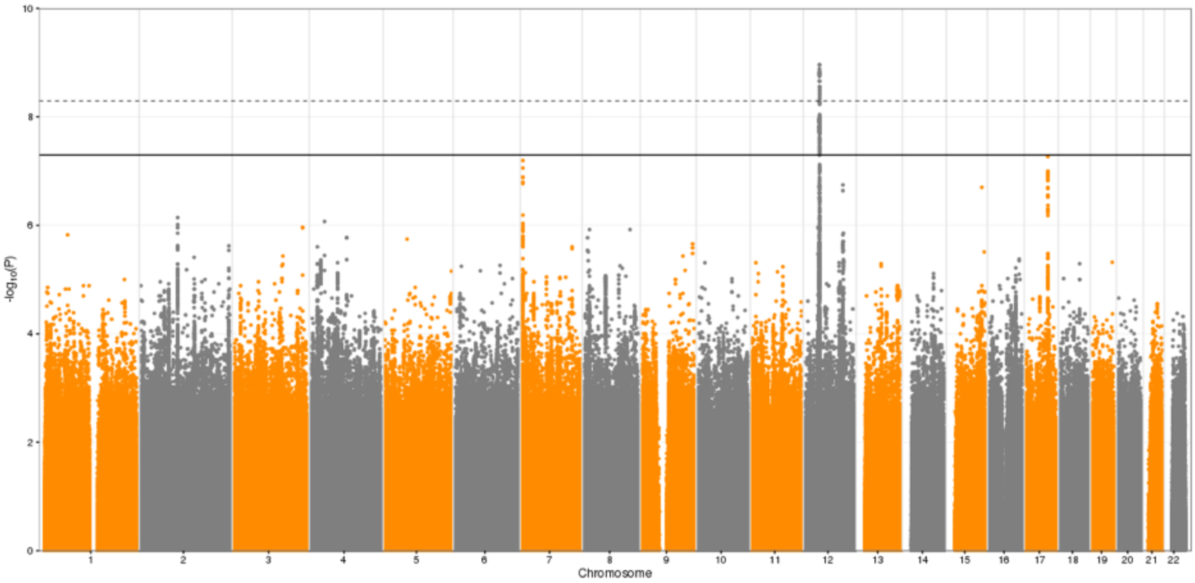
-log10(P)
Chromosome
1 2 3 4 5 6 7 8 9 10 11 12 13 14 15 16 17 18 19 20 21 22

g)

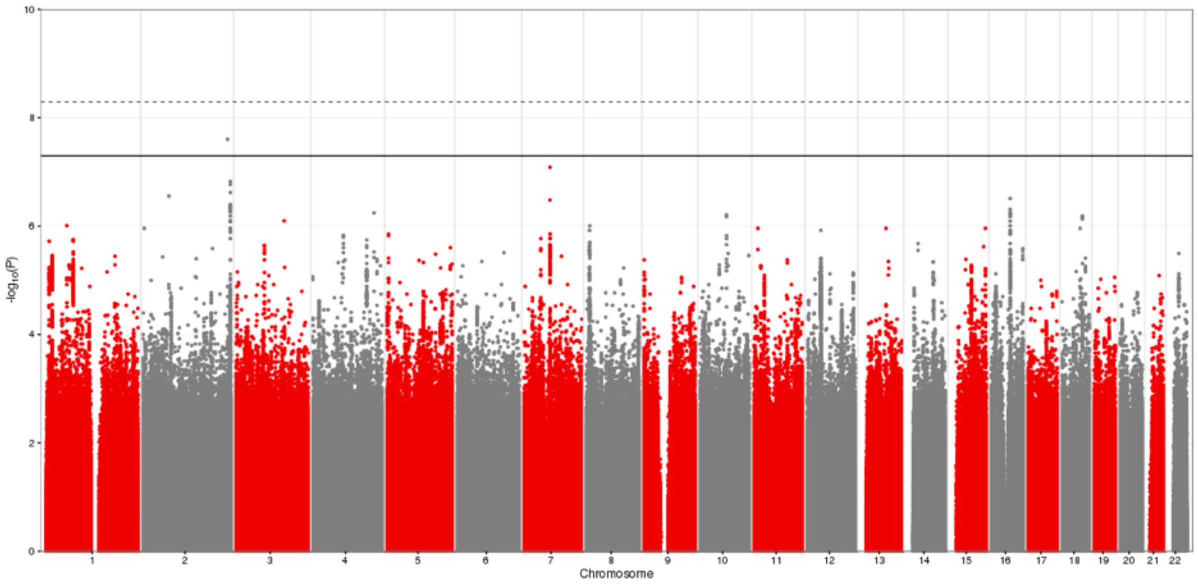

h)

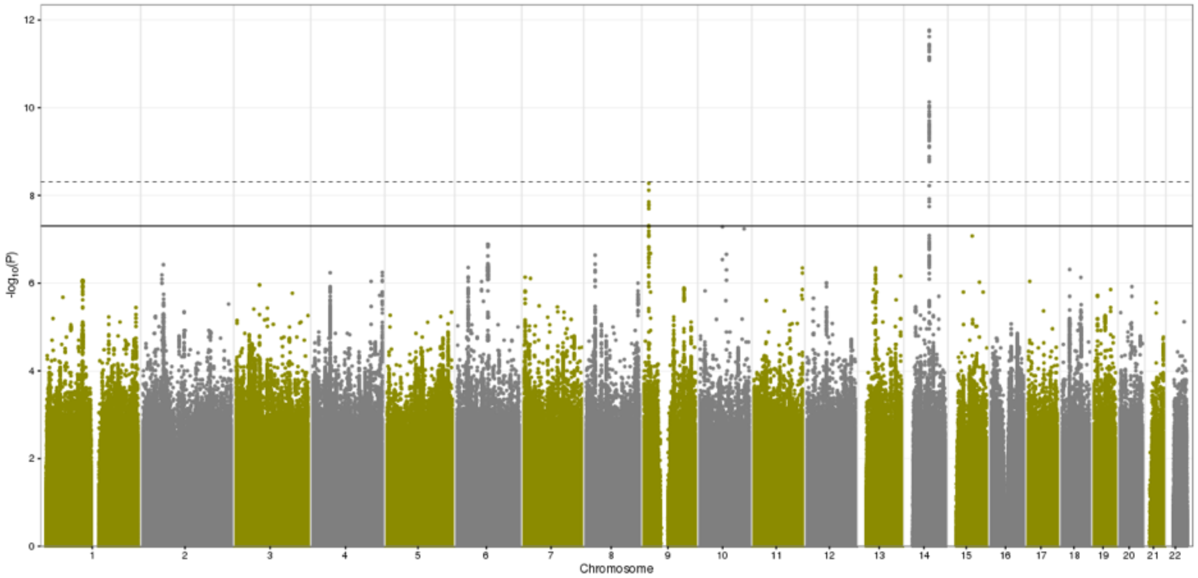

Supplementary Figure 1. Manhattan plots for the eight accelerometer-derived phenotypes. Plots show GWAS results for a) sleep duration, b) sleep duration variability, c) sleep efficiency, d) number of sleep episodes, e) L5 timing, f) M10 timing, g) sleep midpoint and h) diurnal inactivity.

i)

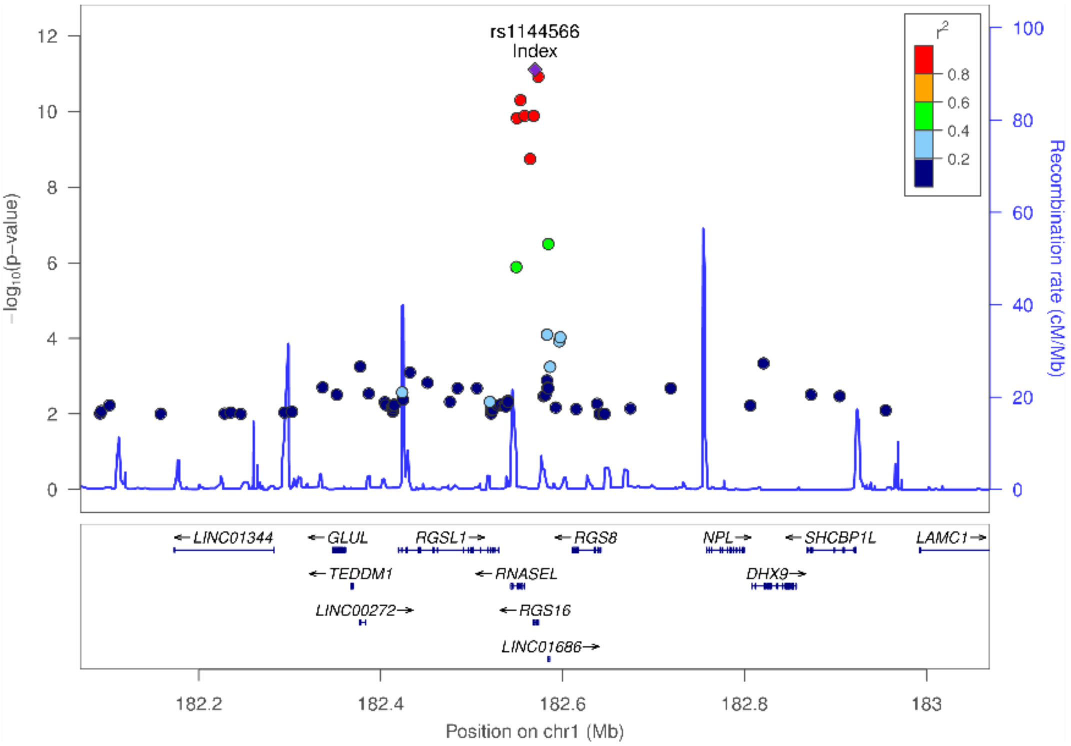
rs1144566
Index
r2
0.8
0.6
0.4
0.2
−log10(p−value)
Recombination rate (cM/Mb)
LINC01344
GLUL
RGSL1
RGS8
NPL
SHCBP1L
LAMC1
TEDDM1
RNASEL
DHX9
LINC00272
RGS16
LINC01686
182.2
182.4
182.6
182.8
183
Position on chr1 (Mb)

ii)

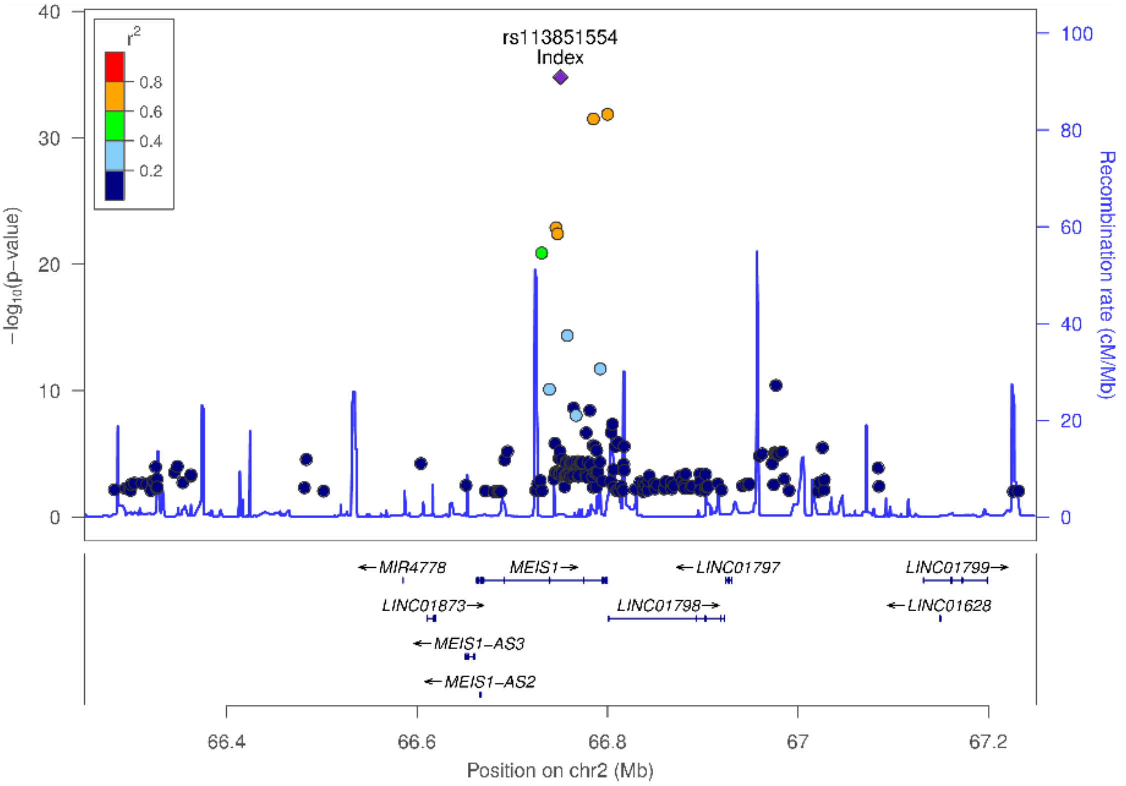
rs113851554
Index
r2
0.8
0.6
0.4
0.2
−log10(p−value)
Recombination rate (cM/Mb)
MIR4778
MEIS1
LINC01797
LINC01799
LINC01873
LINC01798
LINC01628
MEIS1−AS3
MEIS1−AS2
66.4
66.6
66.8
67
67.2
Position on chr2 (Mb)

iii)

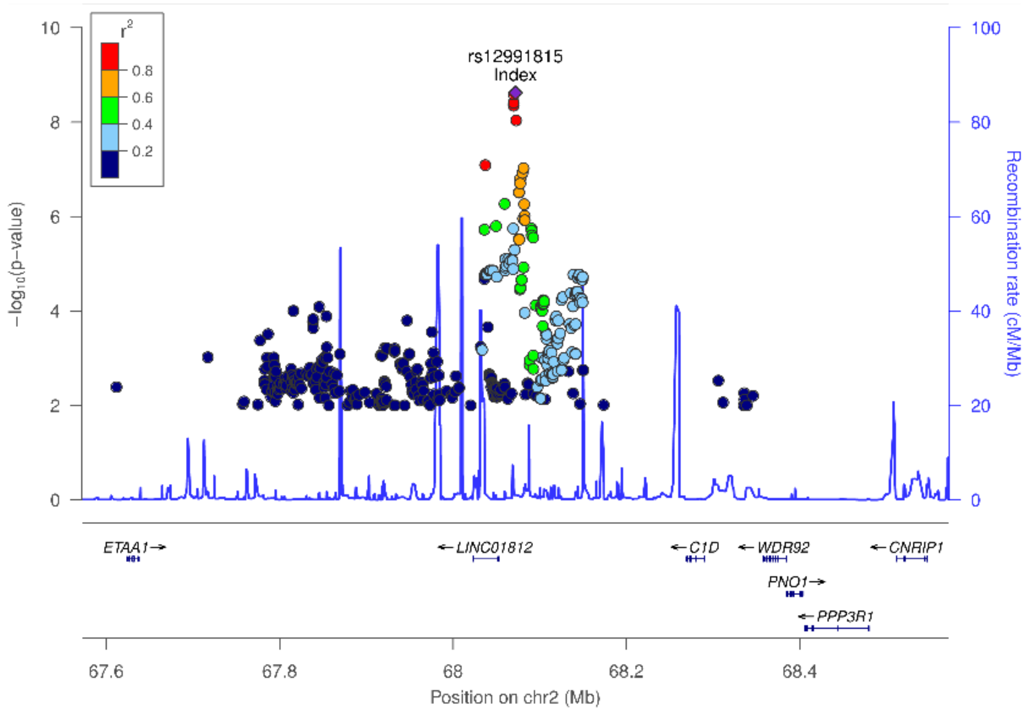

r²
0.8
0.6
0.4
0.2
rs12991815
Index
−log10(p−value)
Recombination rate (cM/Mb)
ETAA1→
←LINC01812
←C1D
←WDR92
←CNRIP1
PNO1→
←PPP3R1
67.6
67.8
68
68.2
68.4
Position on chr2 (Mb)

iv)

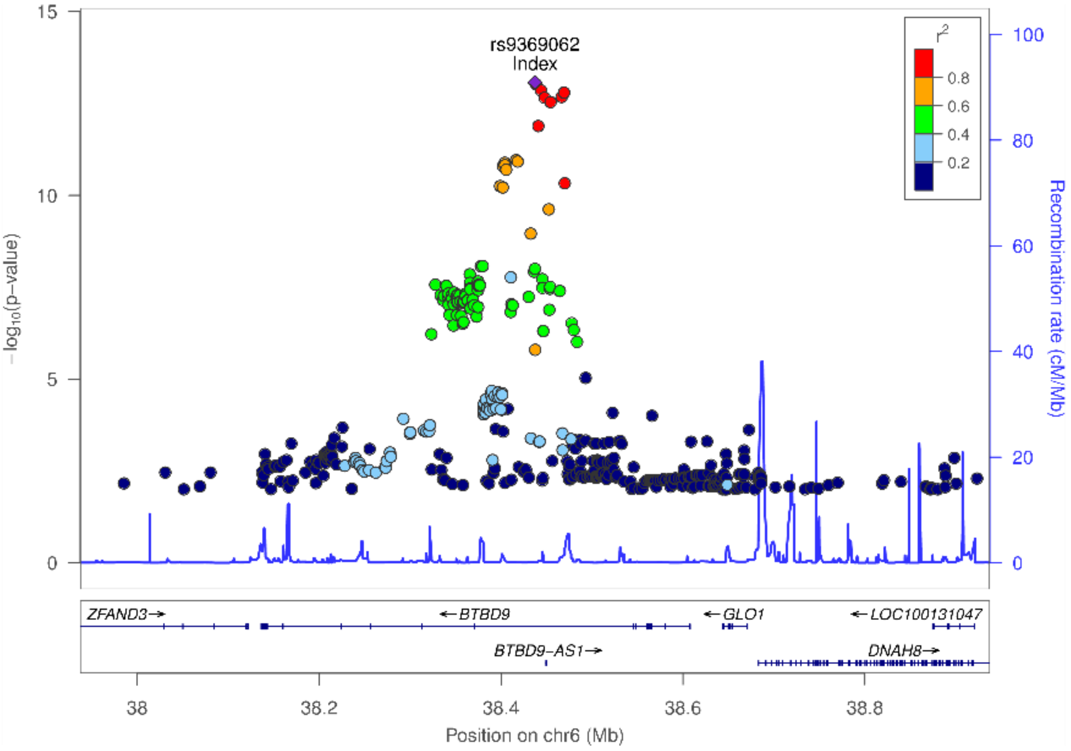

rs9369062
Index
r²
0.8
0.6
0.4
0.2
−log10(p−value)
Recombination rate (cM/Mb)
ZFAND3→
←BTBD9
←GLO1
←LOC100131047
BTBD9−AS1→
DNAH8→
38
38.2
38.4
38.6
38.8
Position on chr6 (Mb)

v)

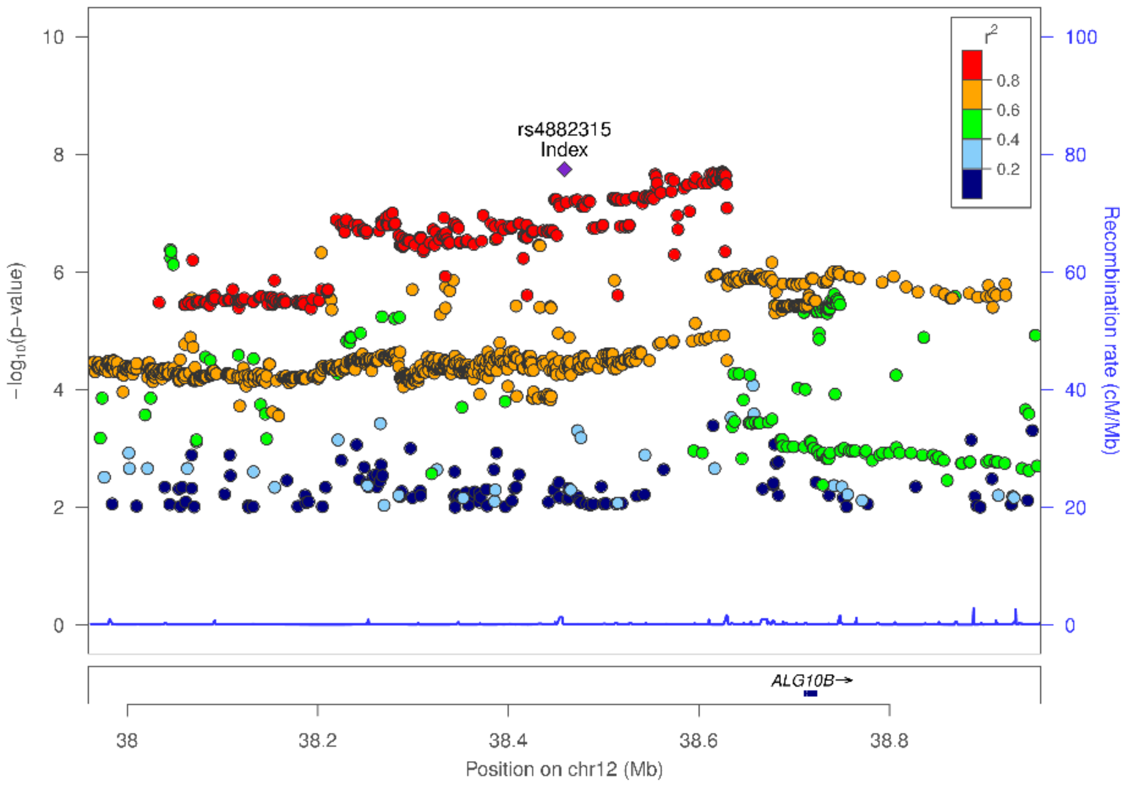

rs4882315
Index
r2
0.8
0.6
0.4
0.2
−log10(p−value)
Recombination rate (cM/Mb)
ALG10B→
38
38.2
38.4
38.6
38.8
Position on chr12 (Mb)

vi)

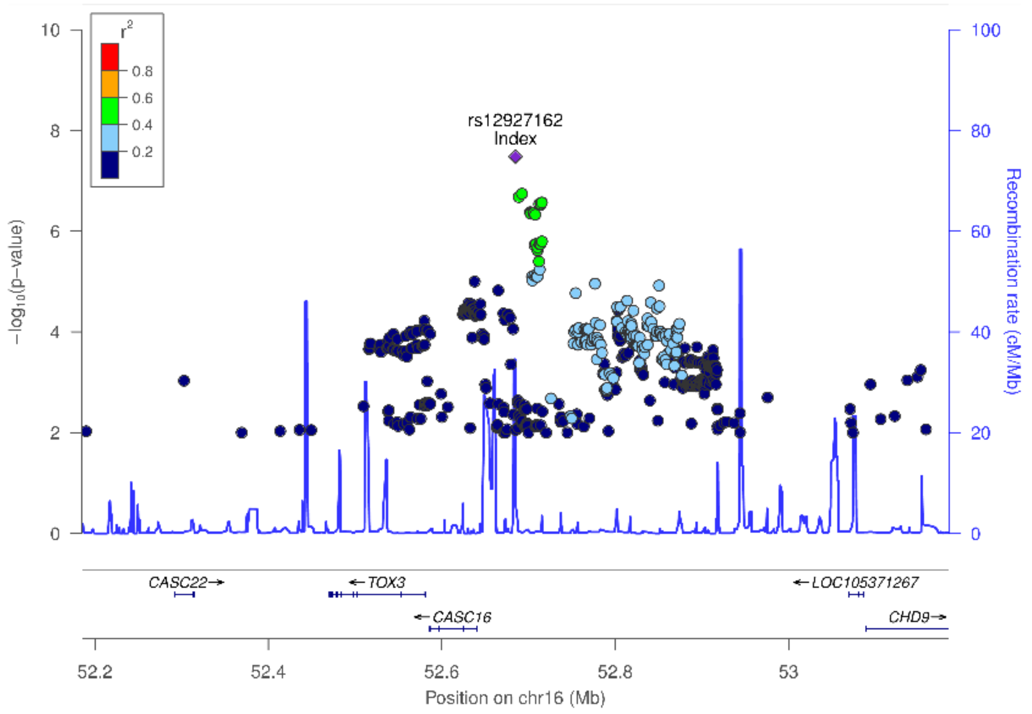

rs12927162
Index
r2
0.8
0.6
0.4
0.2
−log10(p−value)
Recombination rate (cM/Mb)
CASC22→
←TOX3
←CASC16
←LOC105371267
CHD9→
52.2
52.4
52.6
52.8
53
Position on chr16 (Mb)

vii)

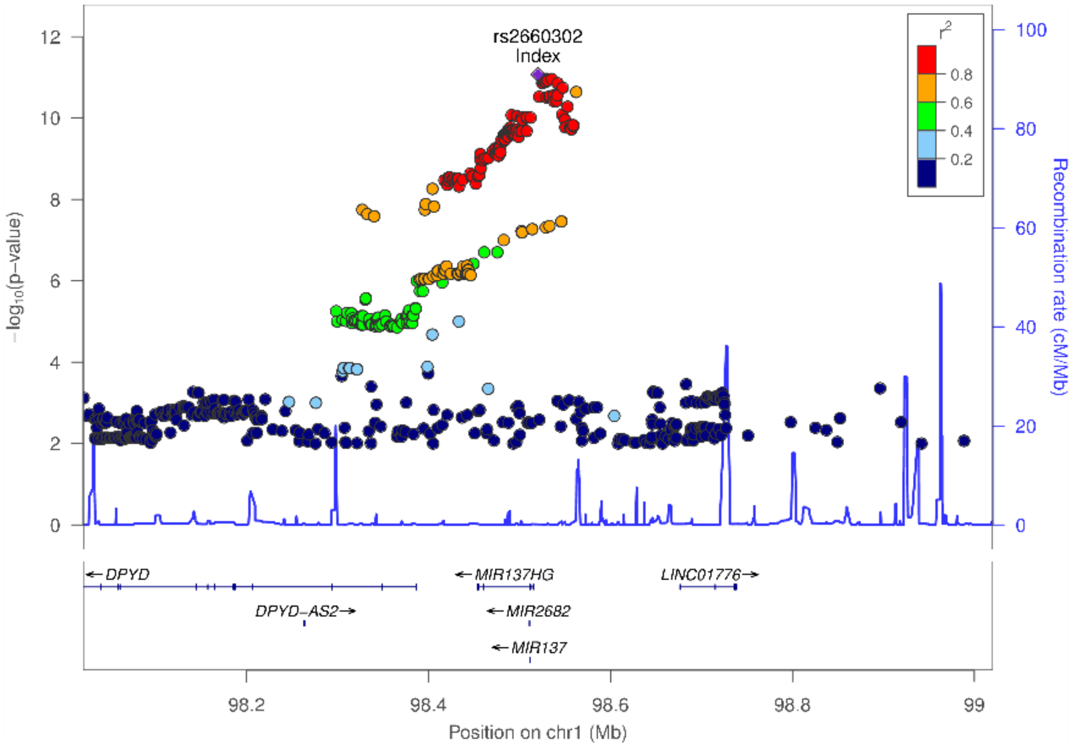
rs2660302
Index
r2
0.8
0.6
0.4
0.2
−log10(p−value)
Recombination rate (cM/Mb)
DPYD
DPYD−AS2
MIR137HG
MIR2682
MIR137
LINC01776
98.2
98.4
98.6
98.8
99
Position on chr1 (Mb)

viii)

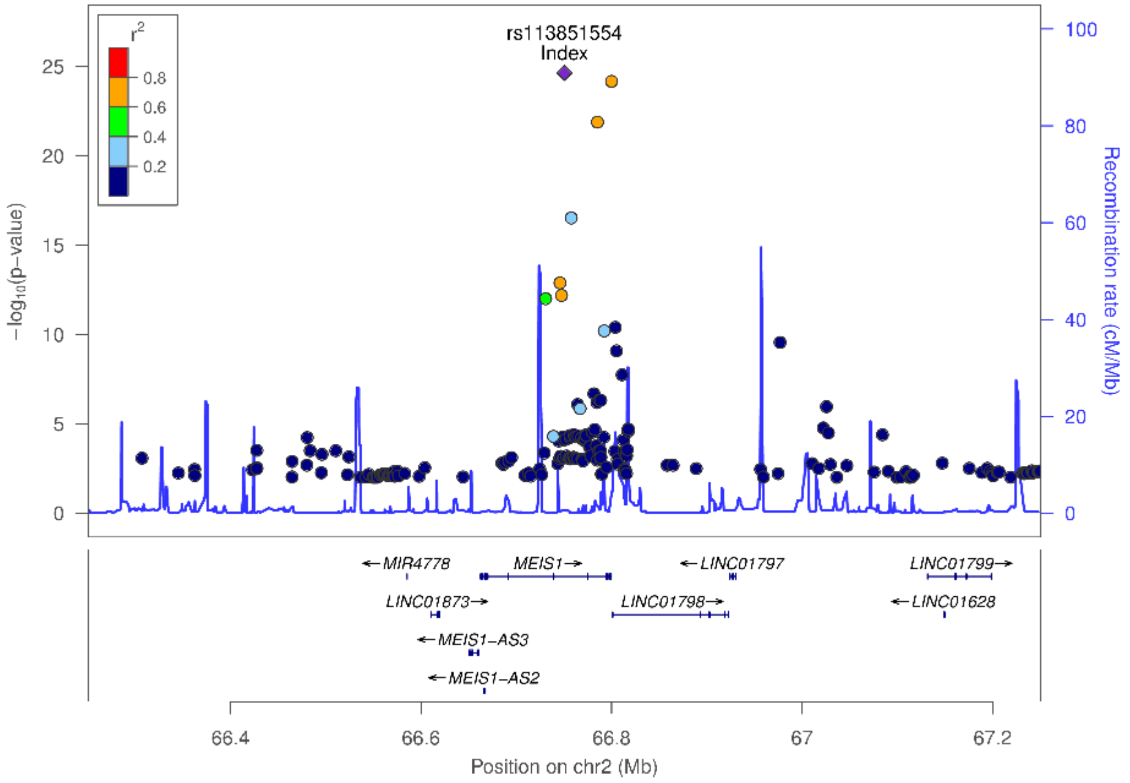
rs113851554
Index
r2
0.8
0.6
0.4
0.2
−log10(p−value)
Recombination rate (cM/Mb)
MIR4778
MEIS1
LINC01797
LINC01799
LINC01873
LINC01798
LINC01628
MEIS1−AS3
MEIS1−AS2
66.4
66.6
66.8
67
67.2
Position on chr2 (Mb)

ix)

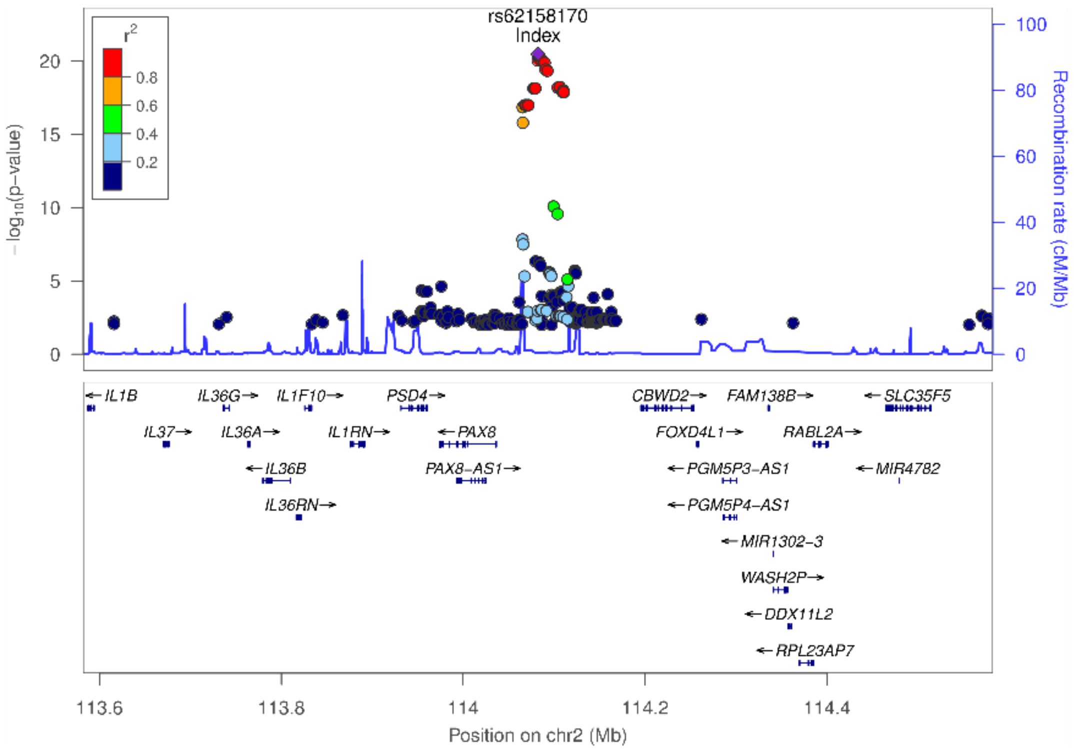
rs62158170
Index
r2
0.8
0.6
0.4
0.2
−log10(p−value)
Recombination rate (cM/Mb)
IL1B
IL36G
IL1F10
PSD4
CBWD2
FAM138B
SLC35F5
IL37
IL36A
IL1RN
PAX8
FOXD4L1
RABL2A
IL36B
PAX8−AS1
PGM5P3−AS1
MIR4782
IL36RN
PGM5P4−AS1
MIR1302−3
WASH2P
DDX11L2
RPL23AP7
113.6
113.8
114
114.2
114.4
Position on chr2 (Mb)

x)

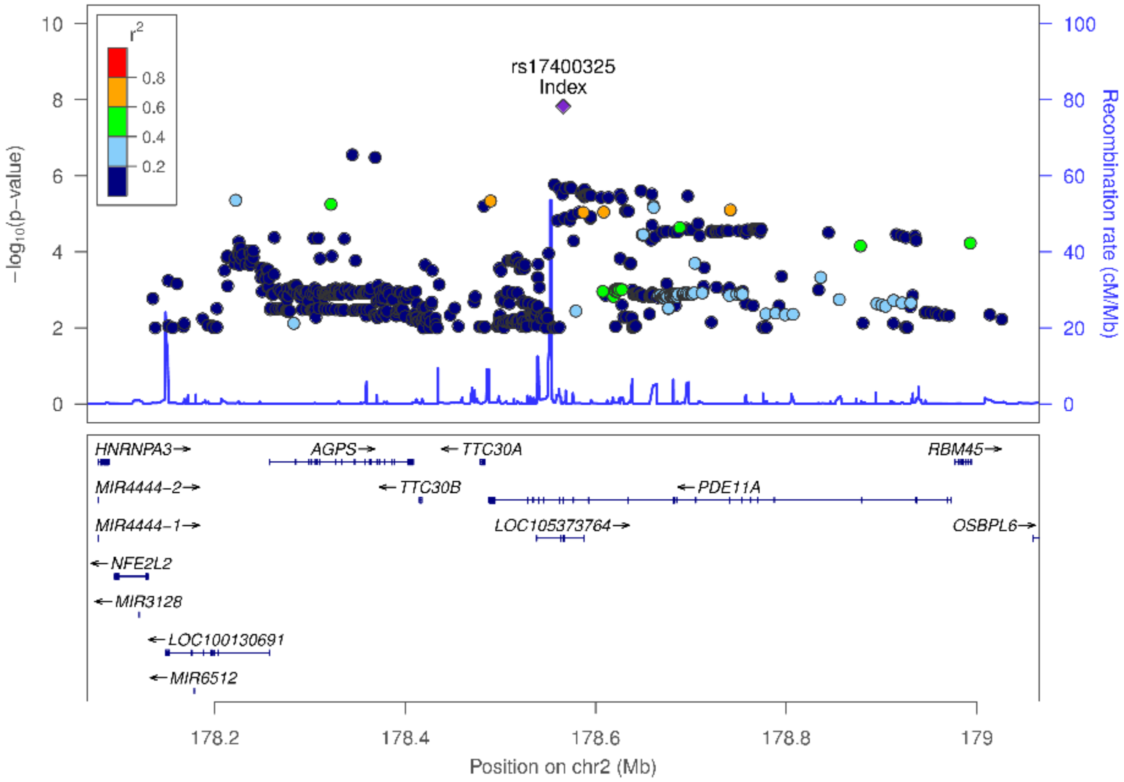
rs17400325
Index
r2
0.8
0.6
0.4
0.2
−log10(p−value)
Recombination rate (cM/Mb)
HNRNPA3
AGPS
TTC30A
RBM45
MIR4444−2
TTC30B
PDE11A
MIR4444−1
LOC105373764
OSBPL6
NFE2L2
MIR3128
LOC100130691
MIR6512
178.2
178.4
178.6
178.8
179
Position on chr2 (Mb)

xi)

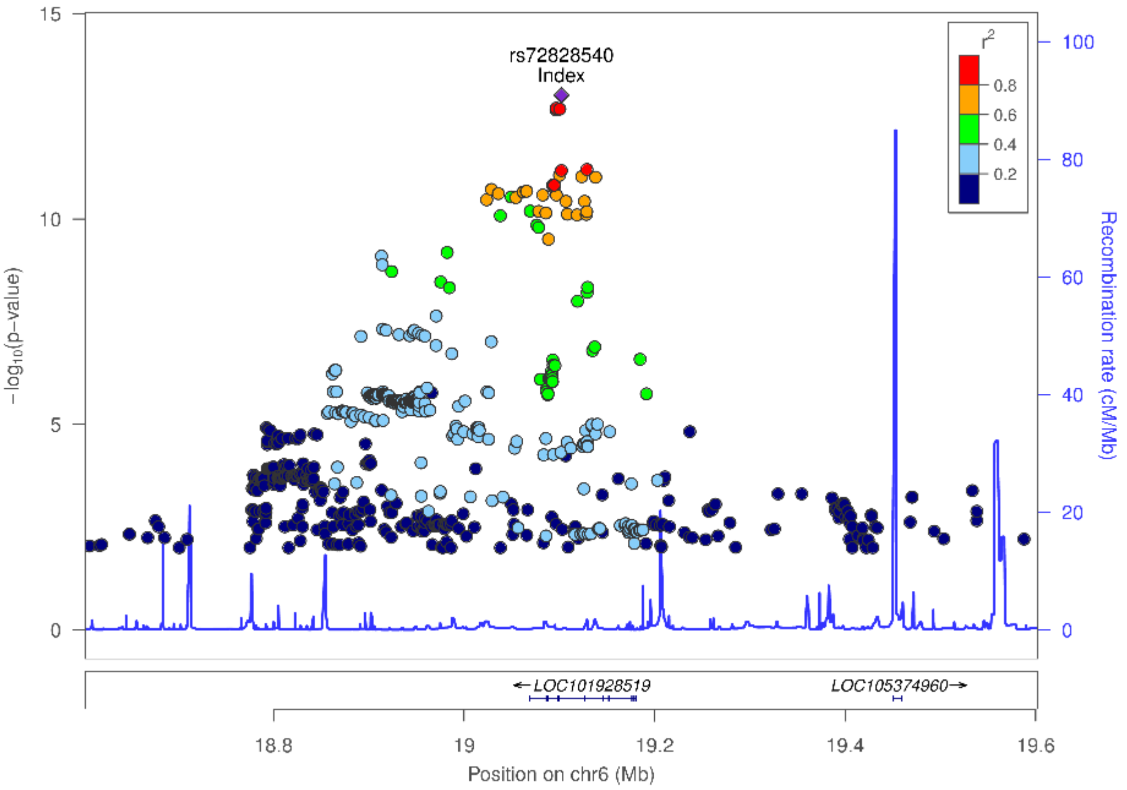
rs72828540
Index
r2
0.8
0.6
0.4
0.2
−log10(p−value)
Recombination rate (cM/Mb)
LOC101928519
LOC105374960
18.8
19
19.2
19.4
19.6
Position on chr6 (Mb)

xii)

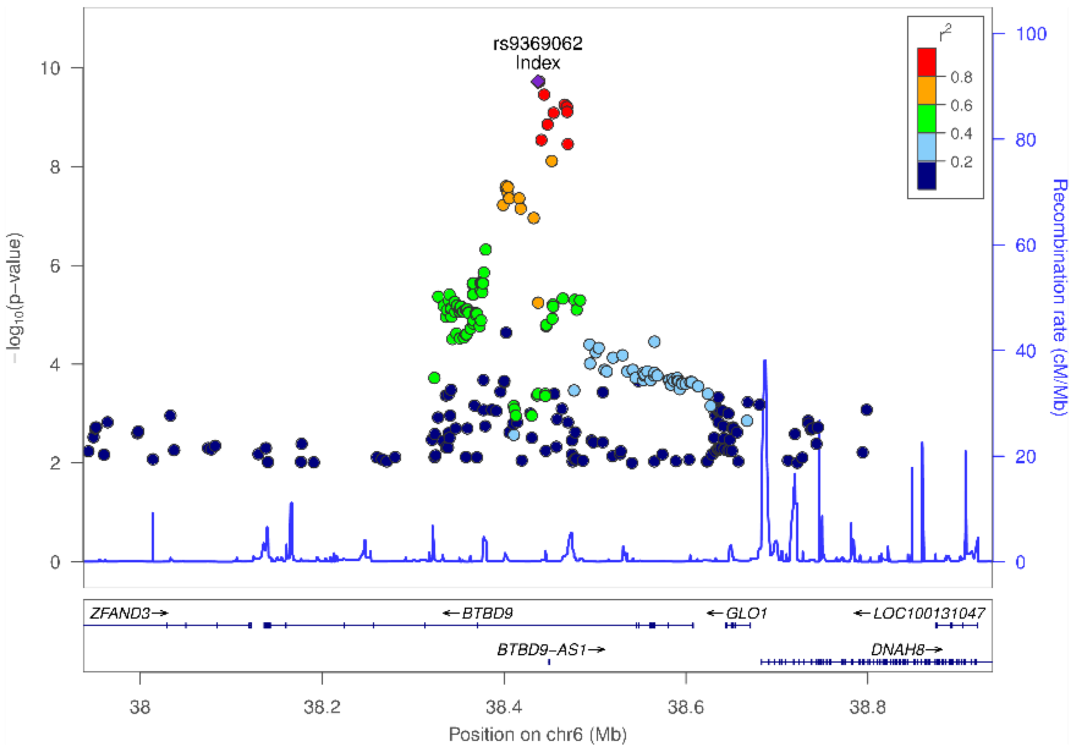
rs9369062
Index
r2
0.8
0.6
0.4
0.2
−log10(p−value)
Recombination rate (cM/Mb)
ZFAND3
BTBD9
GLO1
LOC100131047
BTBD9−AS1
DNAH8
38
38.2
38.4
38.6
38.8
Position on chr6 (Mb)

xiii)

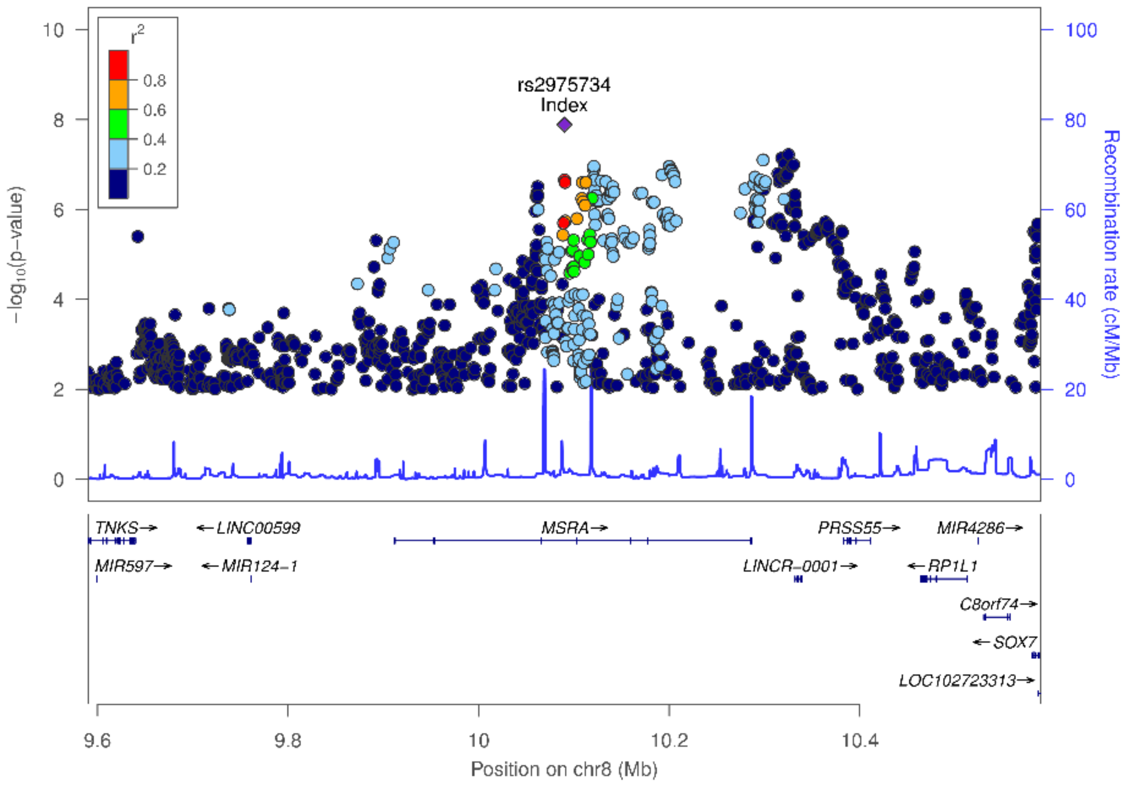
rs2975734
Index
r2
0.8
0.6
0.4
0.2
−log10(p−value)
Recombination rate (cM/Mb)
TNKS→
←LINC00599
MSRA→
PRSS55→
MIR4286→
MIR597→
←MIR124−1
LINCR−0001→
←RP1L1
C8orf74→
←SOX7
LOC102723313→
9.6
9.8
10
10.2
10.4
Position on chr8 (Mb)

xiv)

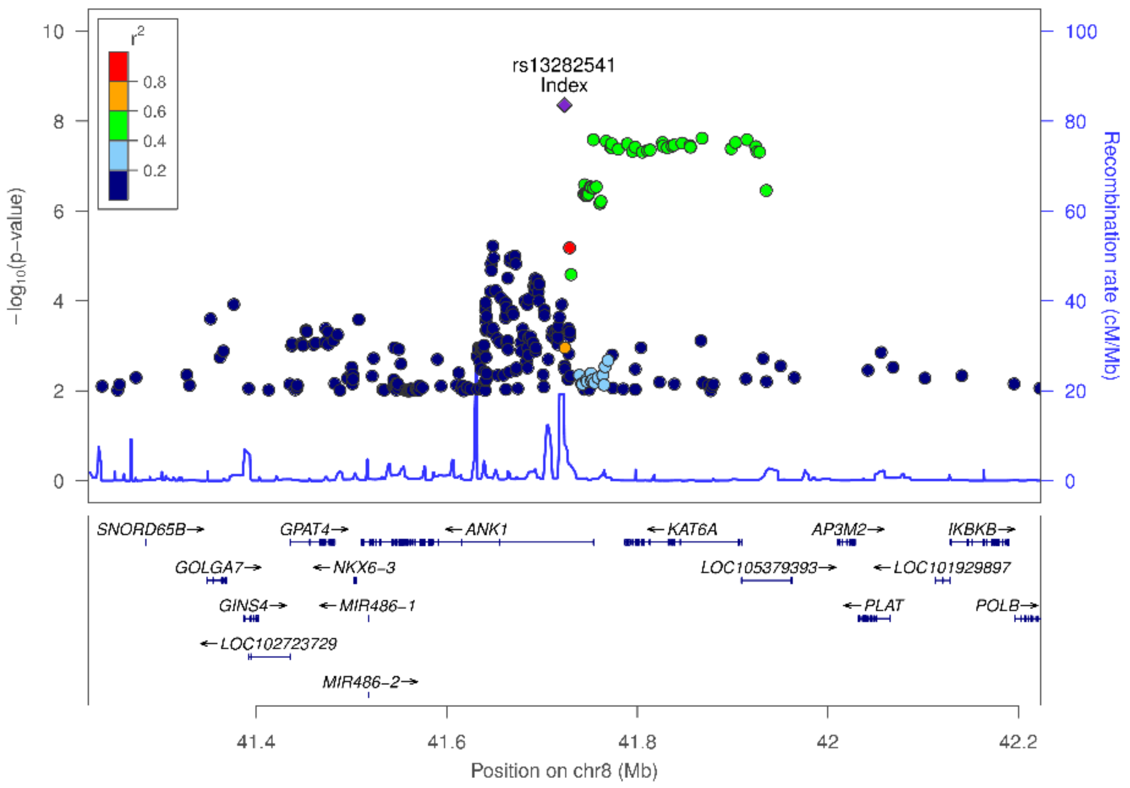
rs13282541
Index
r2
0.8
0.6
0.4
0.2
−log10(p−value)
Recombination rate (cM/Mb)
SNORD65B→
GPAT4→
←ANK1
←KAT6A
AP3M2→
IKBKB→
GOLGA7→
←NKX6−3
LOC105379393→
←LOC101929897
GINS4→
←MIR486−1
←PLAT
POLB→
←LOC102723729
MIR486−2→
41.4
41.6
41.8
42
42.2
Position on chr8 (Mb)

xv)

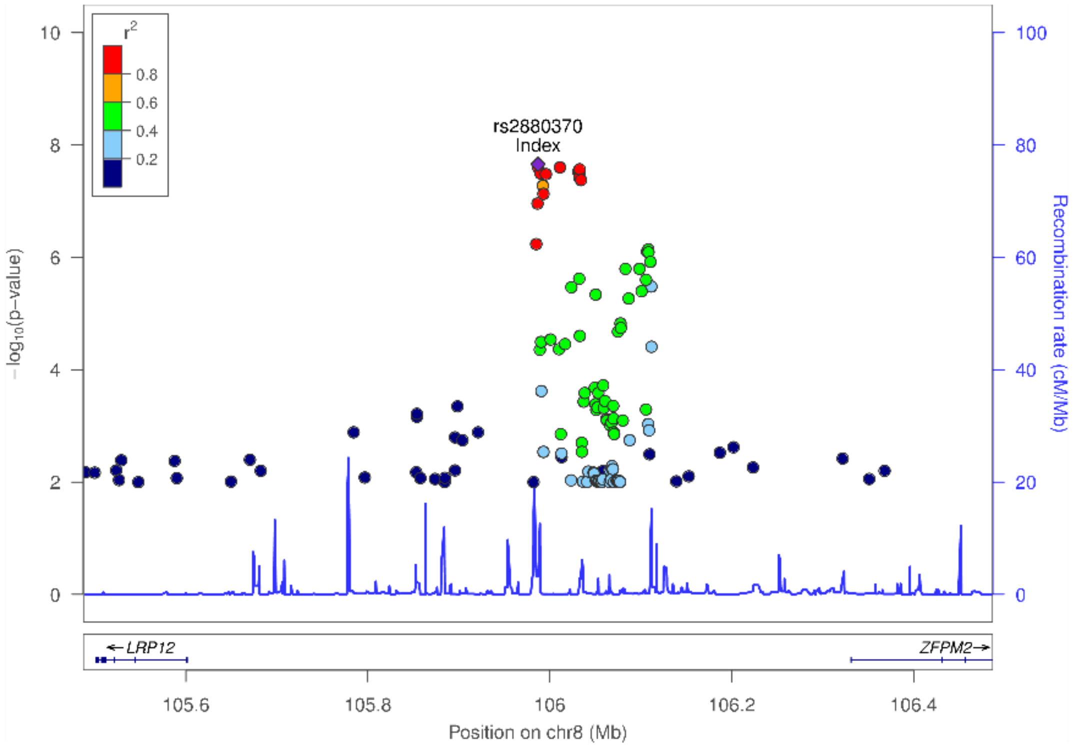

r2
0.8
0.6
0.4
0.2
rs2880370
Index
−log10(p−value)
Recombination rate (cM/Mb)
LRP12
ZFPM2
105.6
105.8
106
106.2
106.4
Position on chr8 (Mb)

xvi)

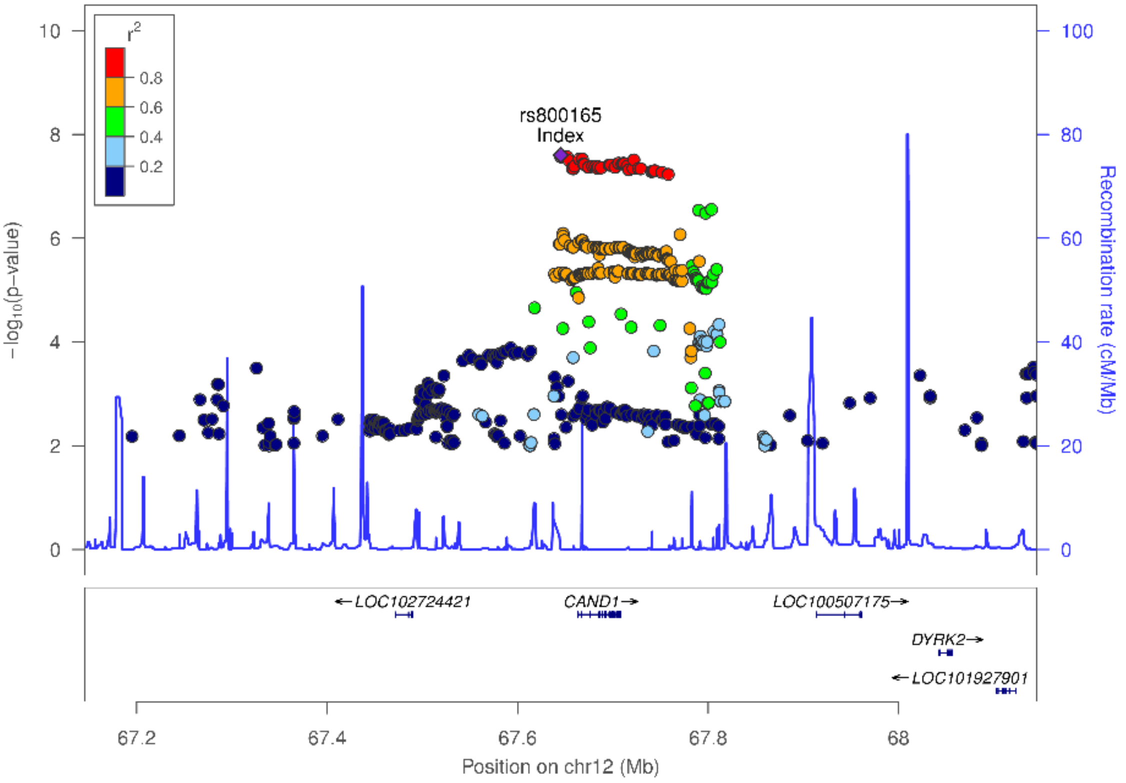

r2
0.8
0.6
0.4
0.2
rs800165
Index
−log10(p−value)
Recombination rate (cM/Mb)
LOC102724421
CAND1
LOC100507175
DYRK2
LOC101927901
67.2
67.4
67.6
67.8
68
Position on chr12 (Mb)

xvii)

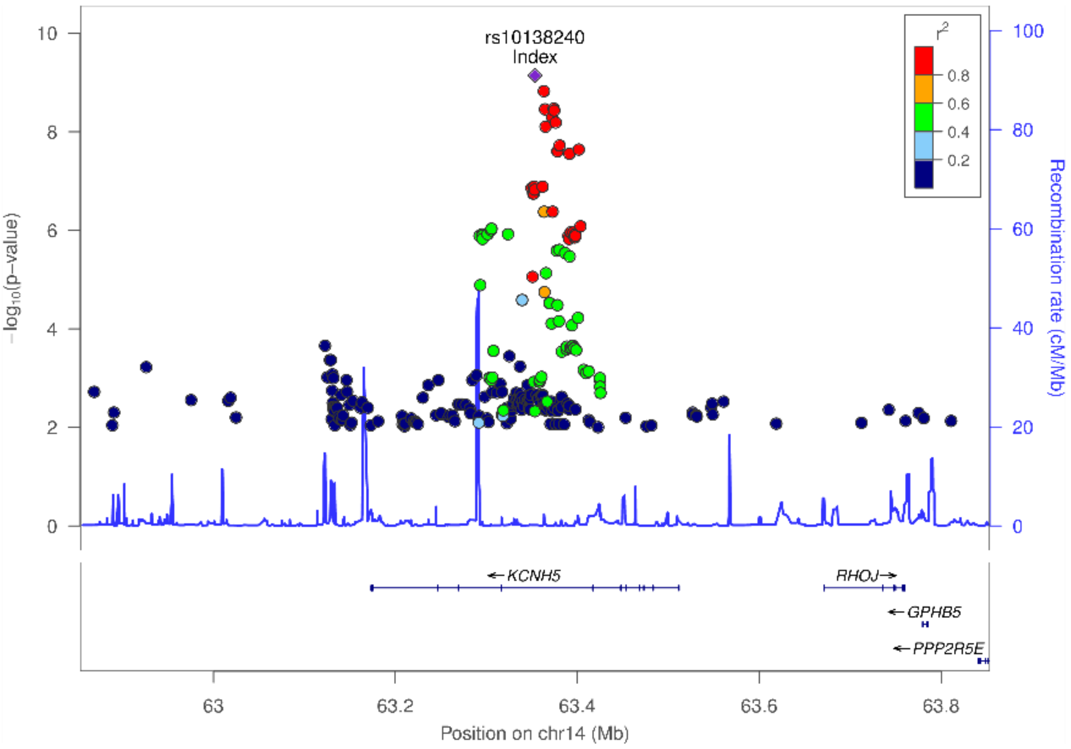
rs10138240
Index
r^2
0.8
0.6
0.4
0.2
−log₁₀(p−value)
Recombination rate (cM/Mb)
KCNH5
RHOJ
GPHB5
PPP2R5E
63
63.2
63.4
63.6
63.8
Position on chr14 (Mb)

xviii)

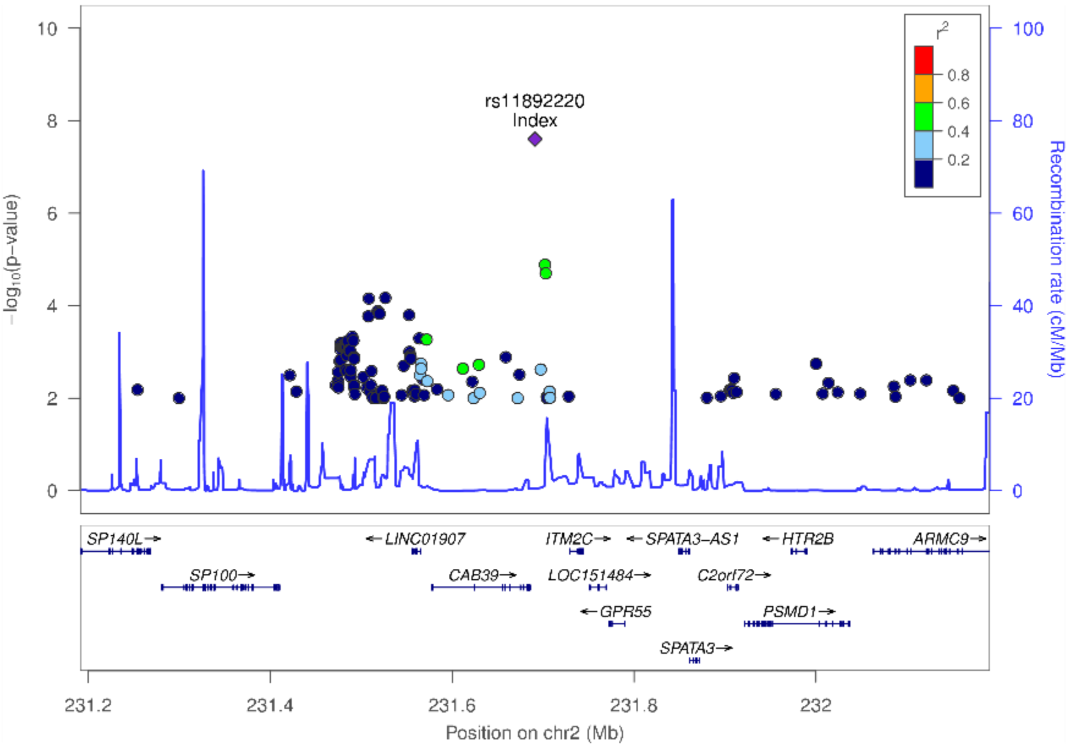
rs11892220
Index
r^2
0.8
0.6
0.4
0.2
−log₁₀(p−value)
Recombination rate (cM/Mb)
SP140L
SP100
LINC01907
CAB39
ITM2C
LOC151484
GPR55
SPATA3-AS1
SPATA3
C2orf72
HTR2B
PSMD1
ARMC9
231.2
231.4
231.6
231.8
232
Position on chr2 (Mb)

xix)

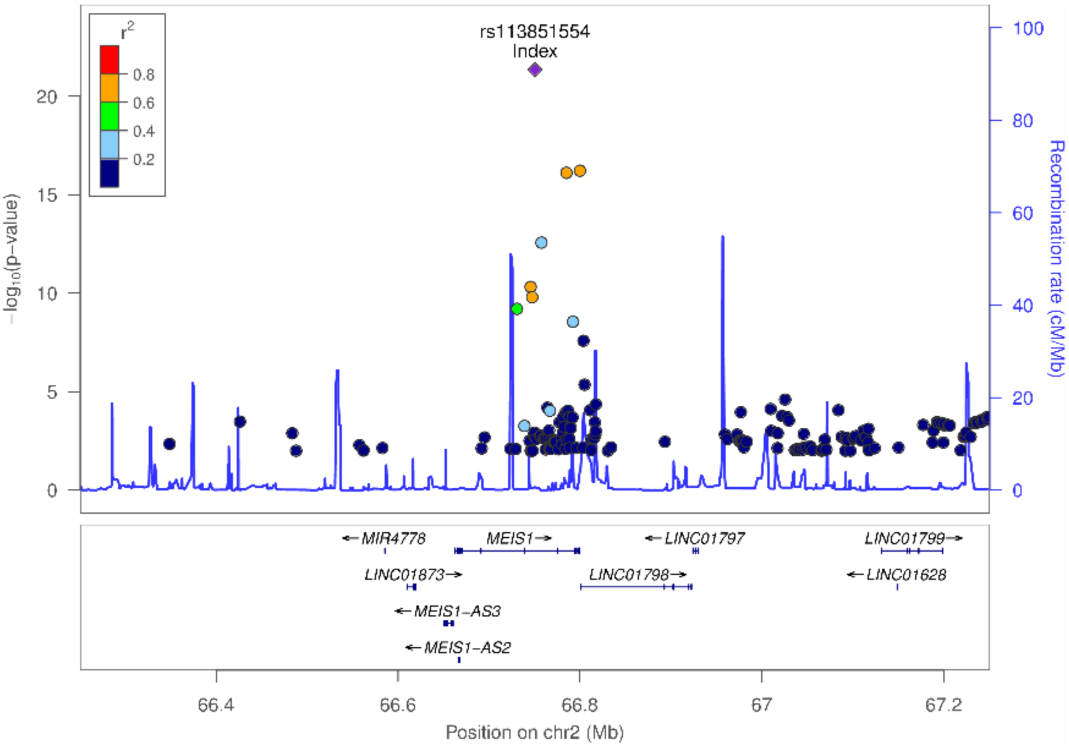

rs113851554
Index
r2
0.8
0.6
0.4
0.2
−log10(p−value)
Recombination rate (cM/Mb)
MIR4778
MEIS1
LINC01797
LINC01799
LINC01873
LINC01798
LINC01628
MEIS1−AS3
MEIS1−AS2
66.4
66.6
66.8
67
67.2
Position on chr2 (Mb)

xx)

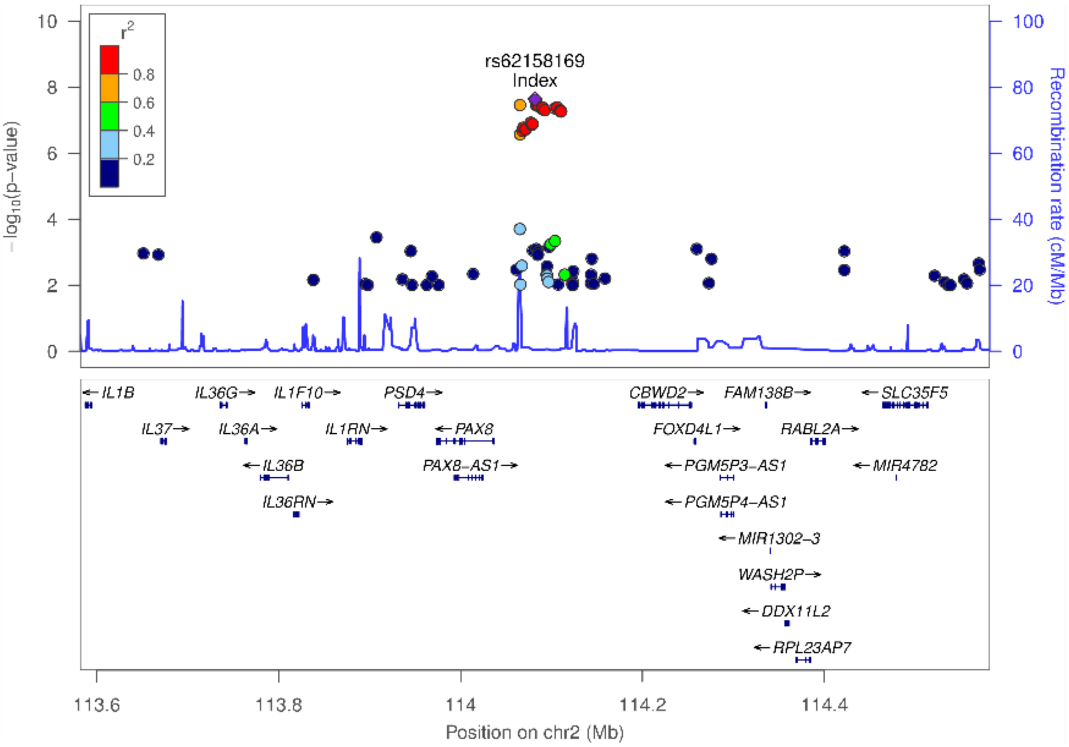

rs62158169
Index
r2
0.8
0.6
0.4
0.2
−log10(p−value)
Recombination rate (cM/Mb)
IL1B
IL36G
IL1F10
PSD4
CBWD2
FAM138B
SLC35F5
IL37
IL36A
IL1RN
PAX8
FOXD4L1
RABL2A
IL36B
PAX8−AS1
PGM5P3−AS1
MIR4782
IL36RN
PGM5P4−AS1
MIR1302−3
WASH2P
DDX11L2
RPL23AP7
113.6
113.8
114
114.2
114.4
Position on chr2 (Mb)

xxi)

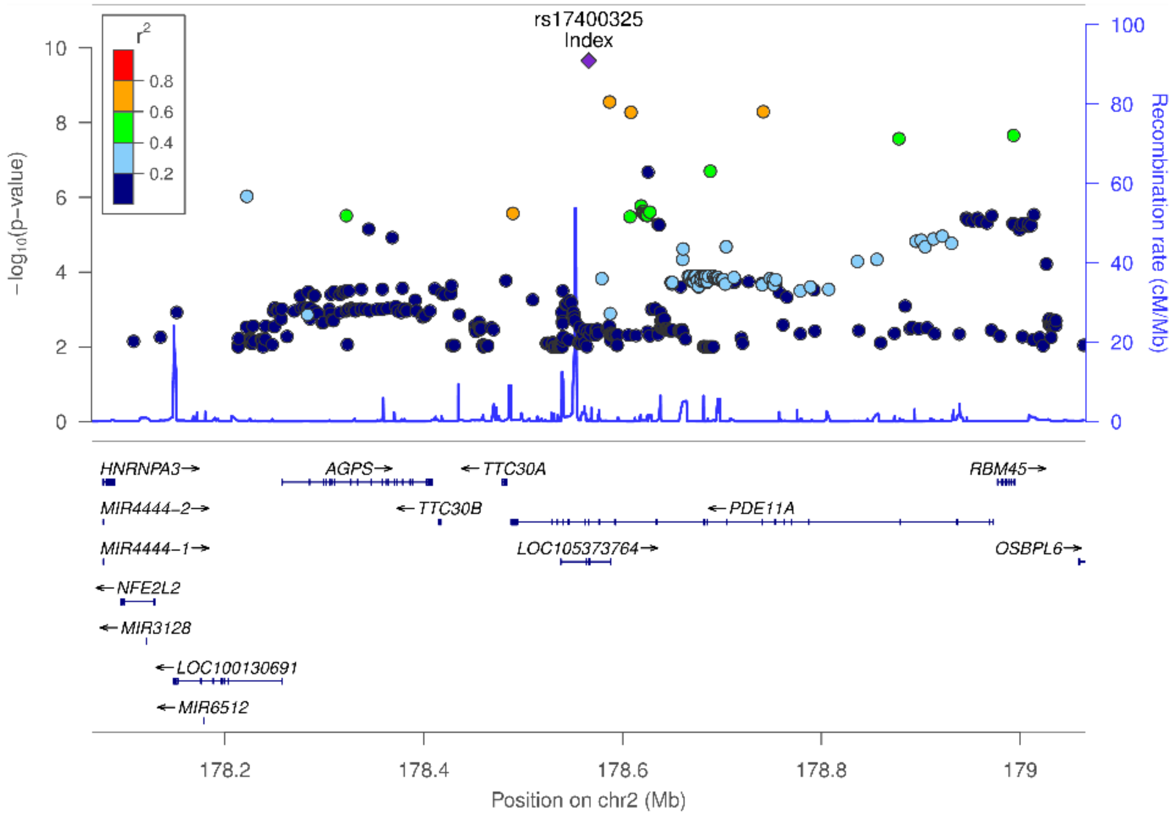
rs17400325
Index
r²
0.8
0.6
0.4
0.2
−log₁₀(p−value)
Recombination rate (cM/Mb)
HNRNPA3
AGPS
TTC30A
RBM45
MIR4444−2
TTC30B
PDE11A
MIR4444−1
LOC105373764
OSBPL6
NFE2L2
MIR3128
LOC100130691
MIR6512
178.2
178.4
178.6
178.8
179
Position on chr2 (Mb)

xxii)

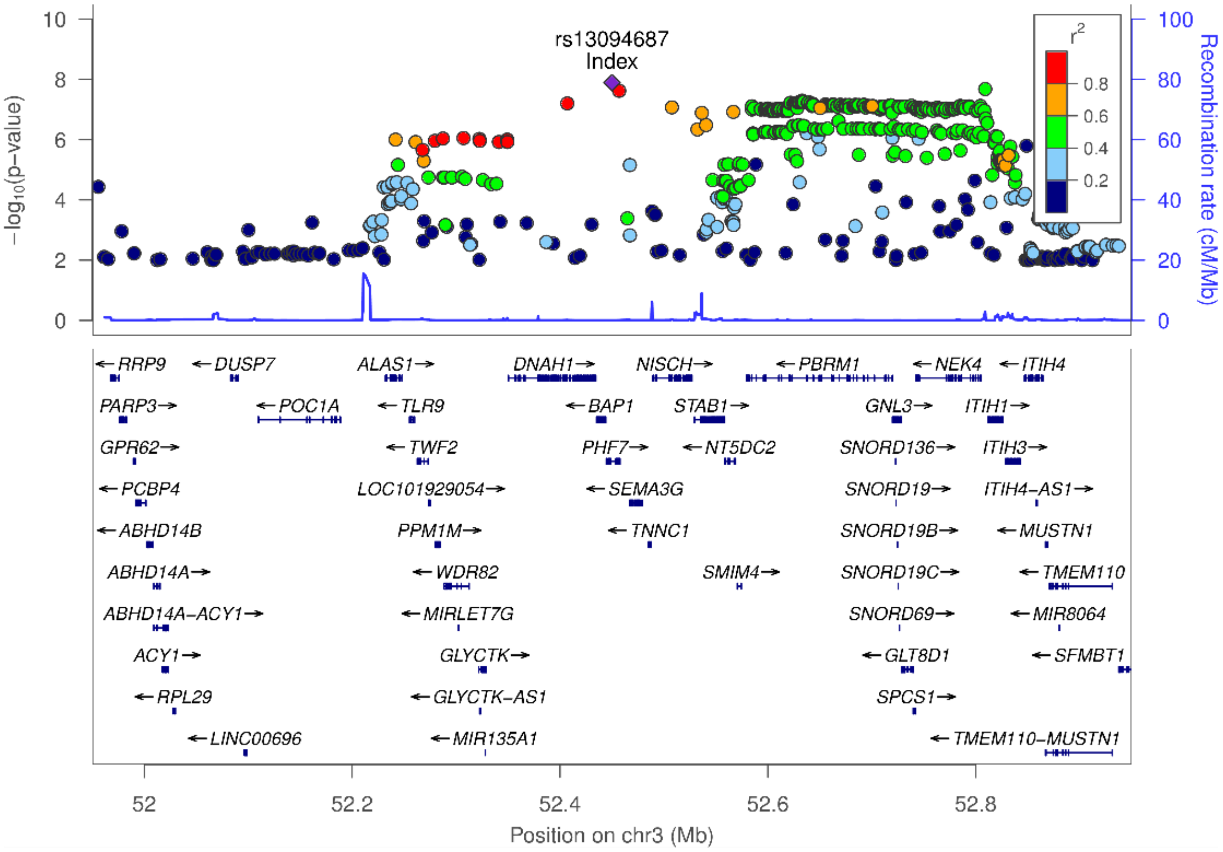
rs13094687
Index
r²
0.8
0.6
0.4
0.2
−log₁₀(p−value)
Recombination rate (cM/Mb)
RRP9
DUSP7
ALAS1
DNAH1
NISCH
PBRM1
NEK4
ITIH4
PARP3
POC1A
TLR9
BAP1
STAB1
GNL3
ITIH1
GPR62
TWF2
PHF7
NT5DC2
SNORD136
ITIH3
PCBP4
LOC101929054
SEMA3G
SNORD19
ITIH4−AS1
ABHD14B
PPM1M
TNNC1
SNORD19B
MUSTN1
ABHD14A
WDR82
SMIM4
SNORD19C
TMEM110
ABHD14A−ACY1
MIRLET7G
SNORD69
MIR8064
ACY1
GLYCTK
GLT8D1
SFMBT1
RPL29
GLYCTK−AS1
SPCS1
LINC00696
MIR135A1
TMEM110−MUSTN1
52
52.2
52.4
52.6
52.8
Position on chr3 (Mb)

xxiii)

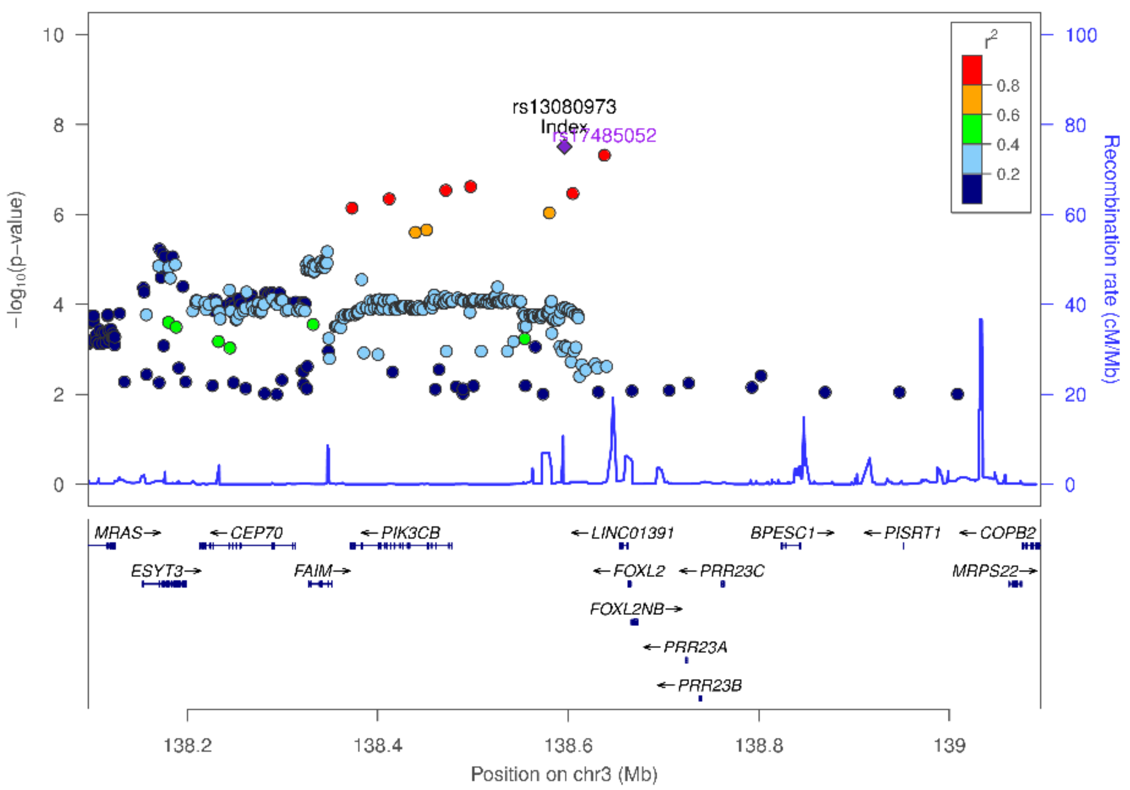

rs13080973
Index
rs17485052
r²
0.8
0.6
0.4
0.2
−log10(p−value)
Recombination rate (cM/Mb)
MRAS→
←CEP70
←PIK3CB
←LINC01391
BPESC1→
←PISRT1
←COPB2
ESYT3→
FAIM→
←FOXL2
←PRR23C
MRPS22→
FOXL2NB→
←PRR23A
←PRR23B
138.2
138.4
138.6
138.8
139
Position on chr3 (Mb)

xxiv)

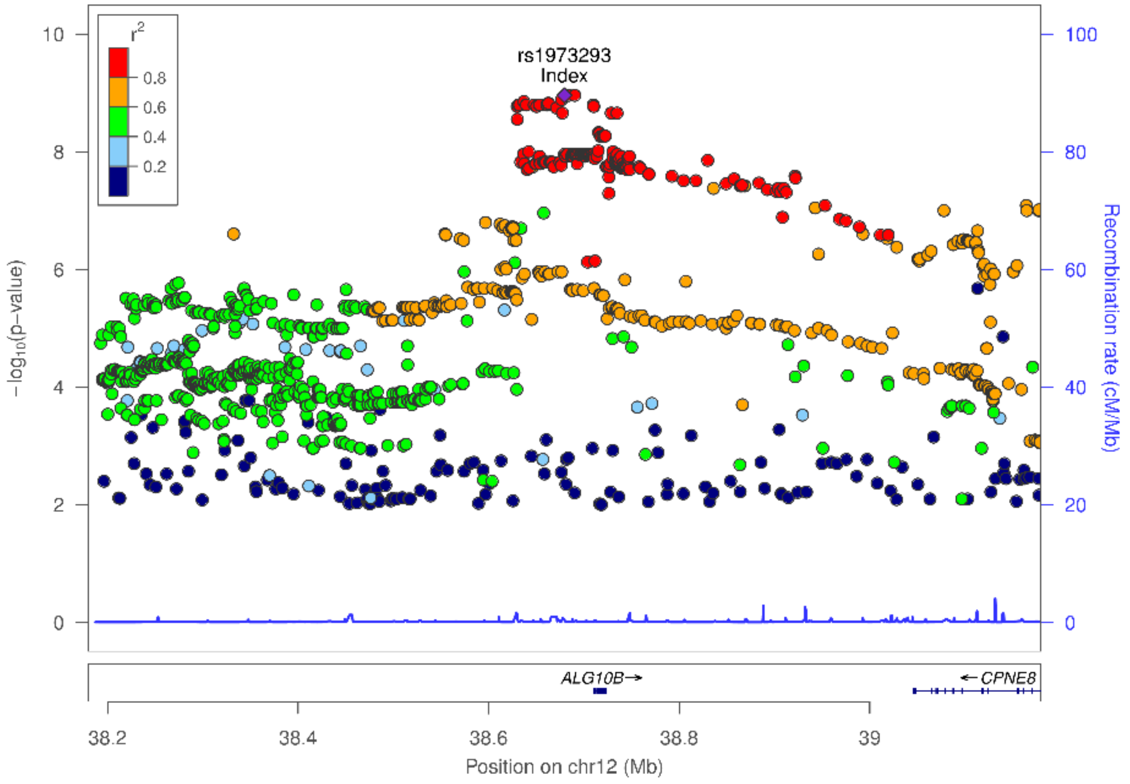

rs1973293
Index
r²
0.8
0.6
0.4
0.2
−log10(p−value)
Recombination rate (cM/Mb)
ALG10B→
←CPNE8
38.2
38.4
38.6
38.8
39
Position on chr12 (Mb)

xxv)

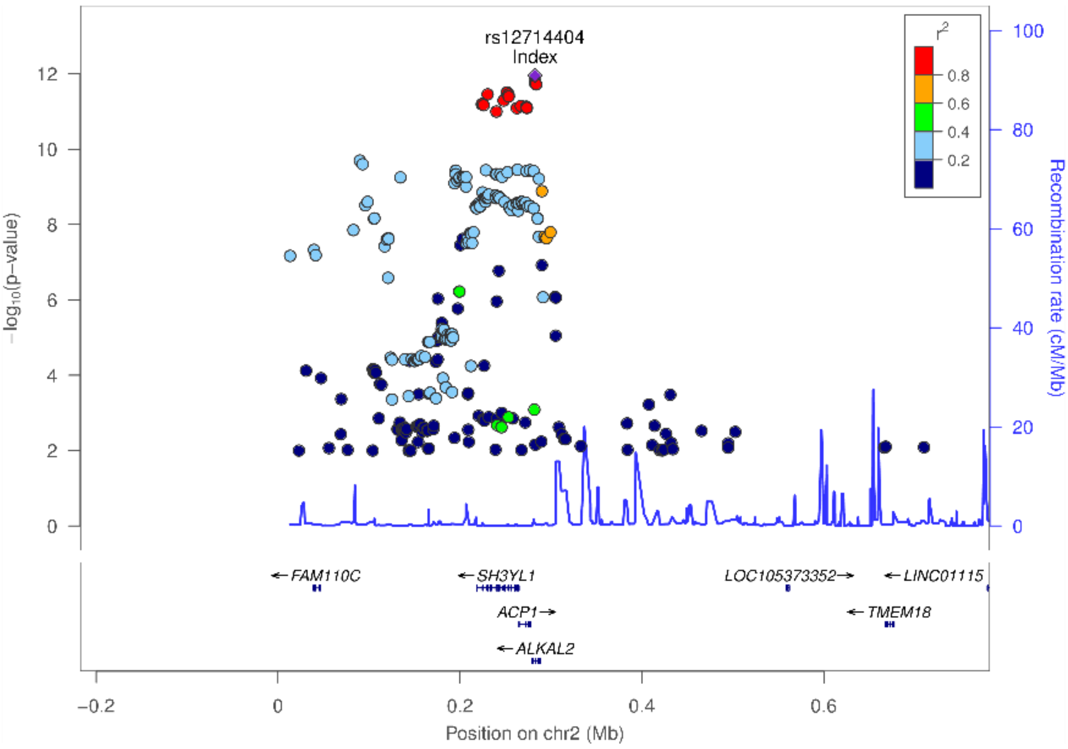
rs12714404
Index
r2
0.8
0.6
0.4
0.2
−log10(p−value)
Recombination rate (cM/Mb)
FAM110C
SH3YL1
ACP1
ALKAL2
LOC105373352
LINC01115
TMEM18
Position on chr2 (Mb)

xxvi)

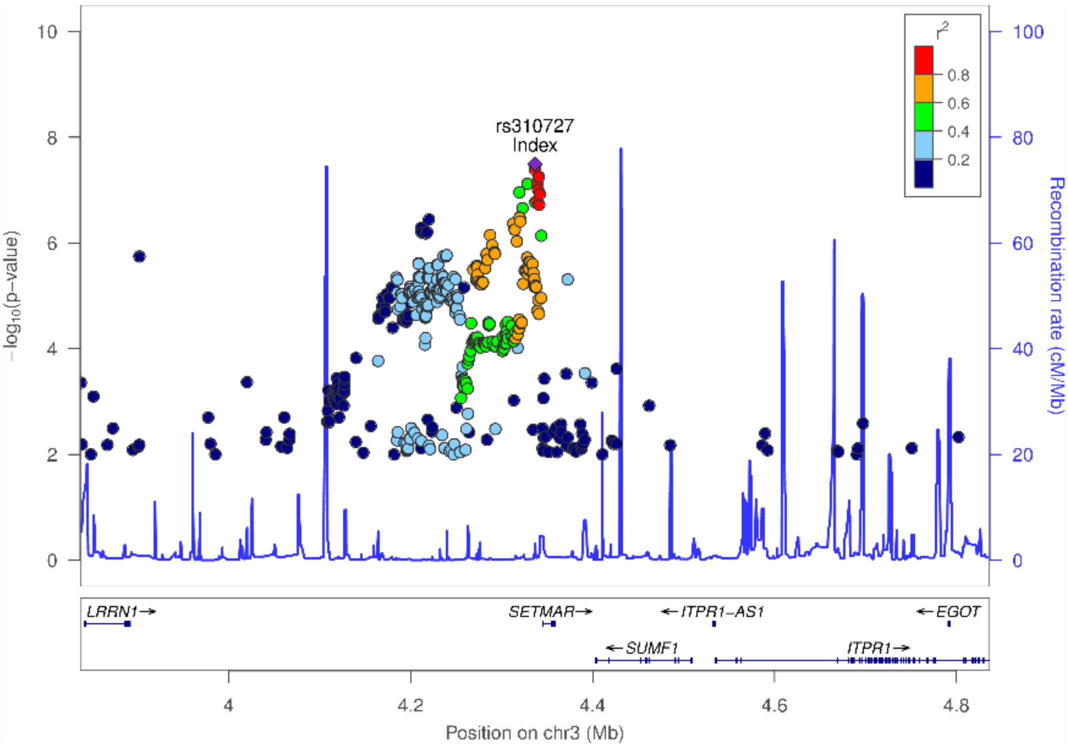
rs310727
Index
r2
0.8
0.6
0.4
0.2
−log10(p−value)
Recombination rate (cM/Mb)
LRRN1
SETMAR
ITPR1−AS1
EGOT
SUMF1
ITPR1
Position on chr3 (Mb)

xxvii)

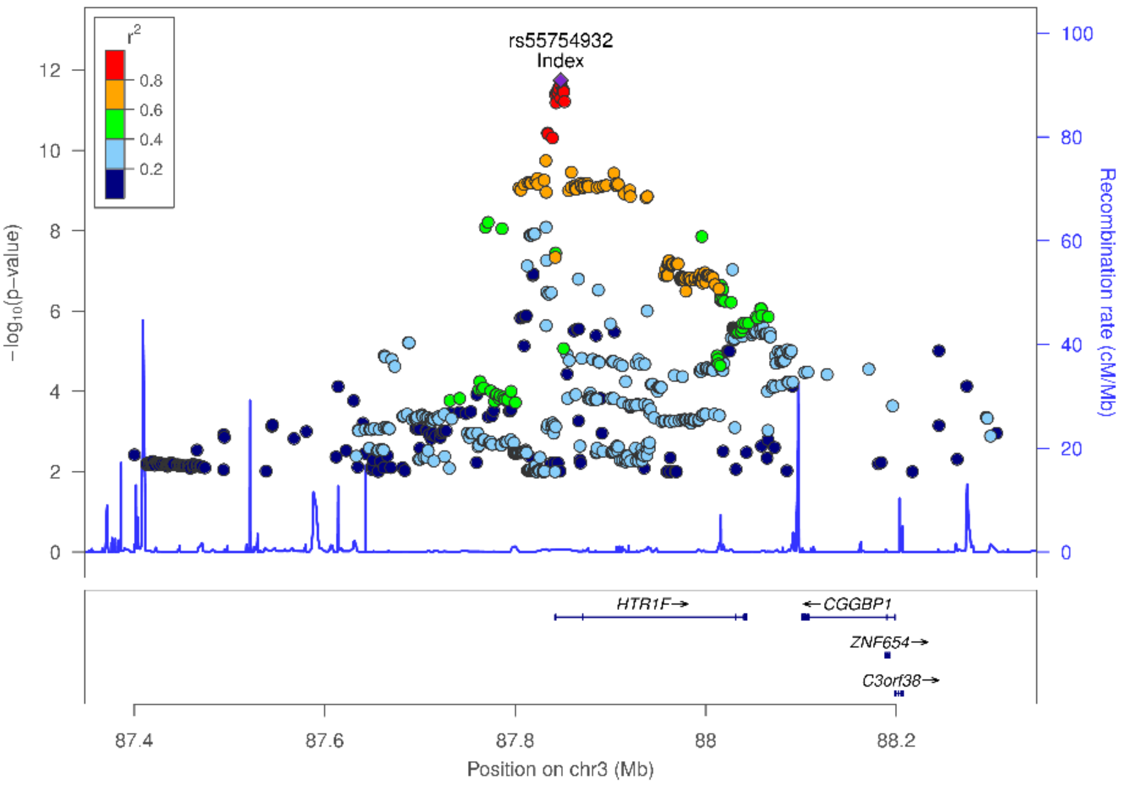

rs55754932
Index
r2
0.8
0.6
0.4
0.2
−log10(p−value)
Recombination rate (cM/Mb)
HTR1F→
←CGGBP1
ZNF654→
C3orf38→
87.4
87.6
87.8
88
88.2
Position on chr3 (Mb)

xxvii)

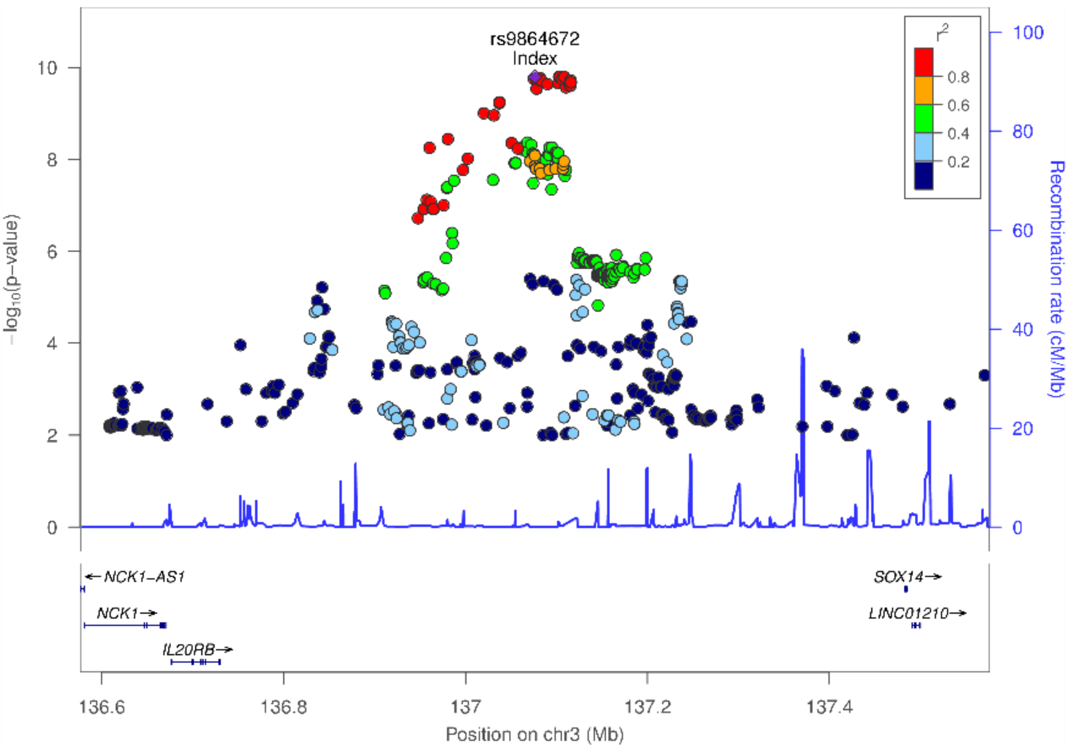

rs9864672
Index
r2
0.8
0.6
0.4
0.2
−log10(p−value)
Recombination rate (cM/Mb)
←NCK1−AS1
NCK1→
IL20RB→
SOX14→
LINC01210→
136.6
136.8
137
137.2
137.4
Position on chr3 (Mb)

xxix)

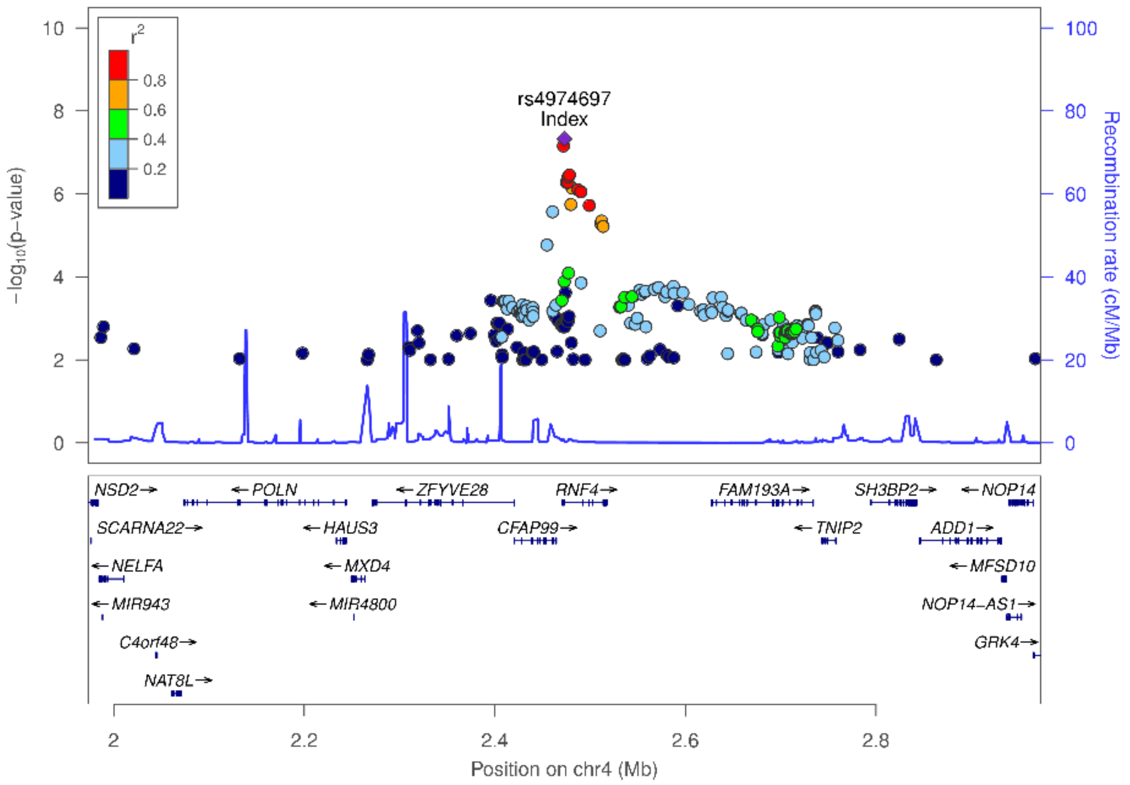
rs4974697
Index
r2
0.8
0.6
0.4
0.2
−log10(p−value)
Recombination rate (cM/Mb)
NSD2→
←POLN
←ZFYVE28
RNF4→
FAM193A→
SH3BP2→
←NOP14
SCARNA22→
←HAUS3
CFAP99→
←TNIP2
ADD1→
←NELFA
←MXD4
←MFSD10
←MIR943
←MIR4800
NOP14−AS1→
C4orf48→
GRK4→
NAT8L→
Position on chr4 (Mb)

xxx)

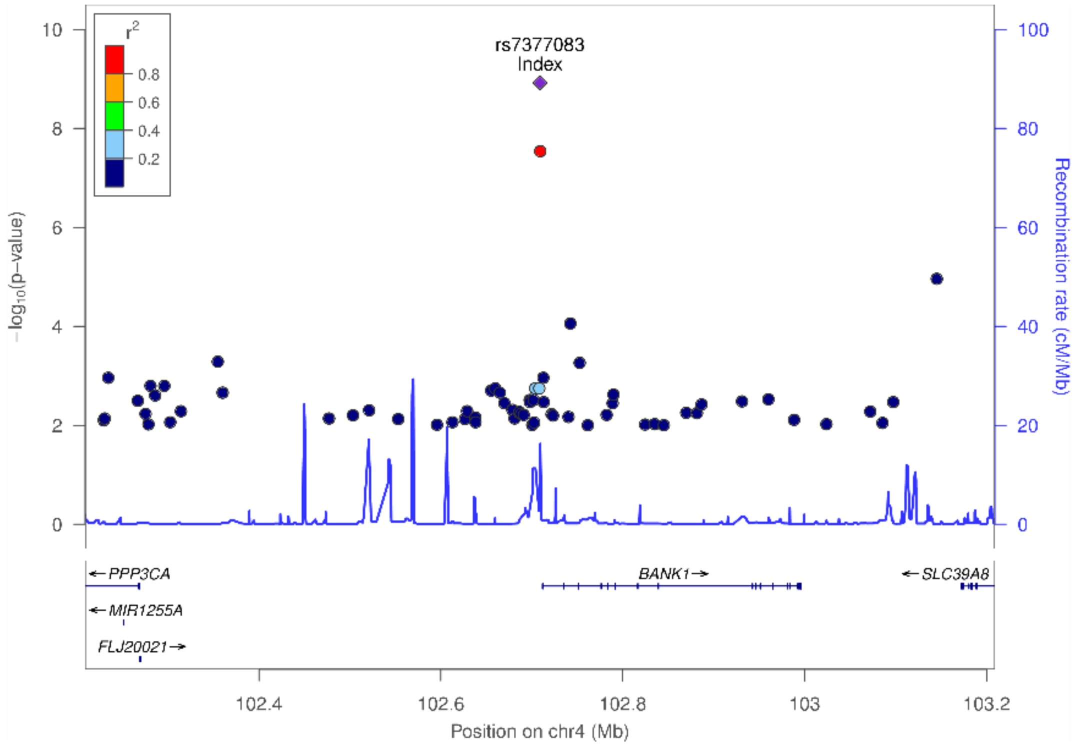
rs7377083
Index
r2
0.8
0.6
0.4
0.2
−log10(p−value)
Recombination rate (cM/Mb)
←PPP3CA
BANK1→
←SLC39A8
←MIR1255A
FLJ20021→
Position on chr4 (Mb)

xxxi)

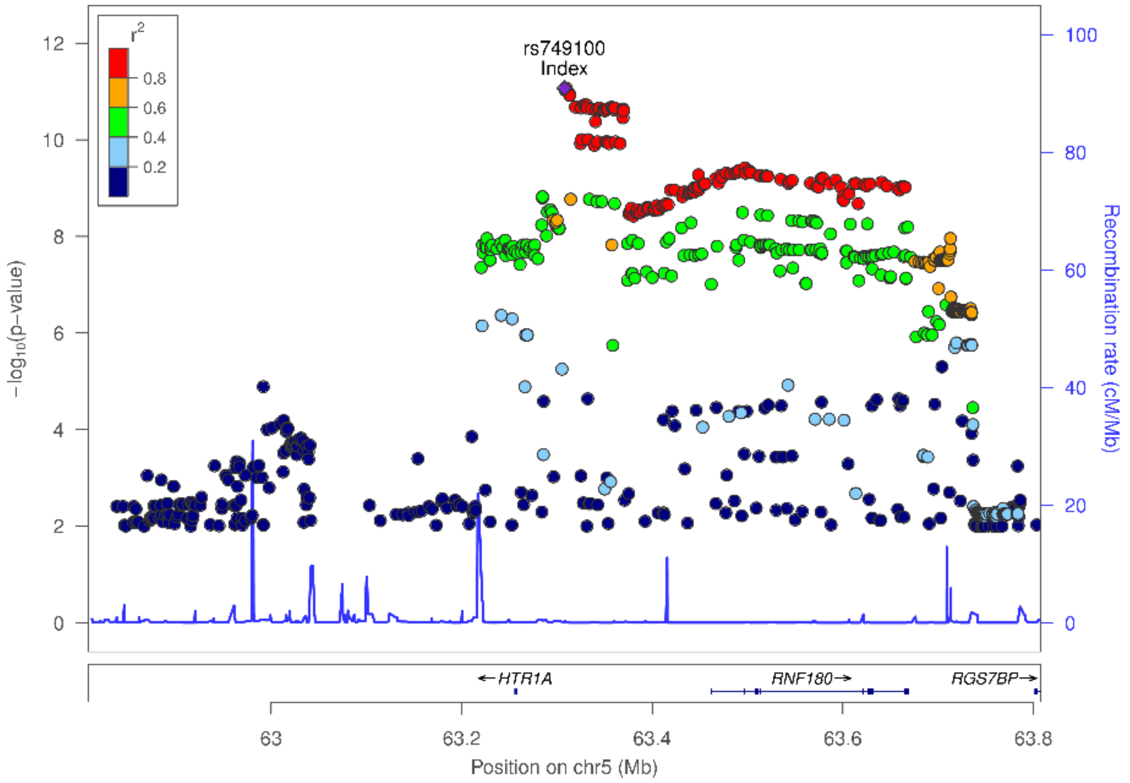
rs749100
Index
r2
0.8
0.6
0.4
0.2
12
10
8
6
4
2
0
−log10(p−value)
100
80
60
40
20
0
Recombination rate (cM/Mb)
HTR1A
RNF180
RGS7BP
63
63.2
63.4
63.6
63.8
Position on chr5 (Mb)

xxxii)

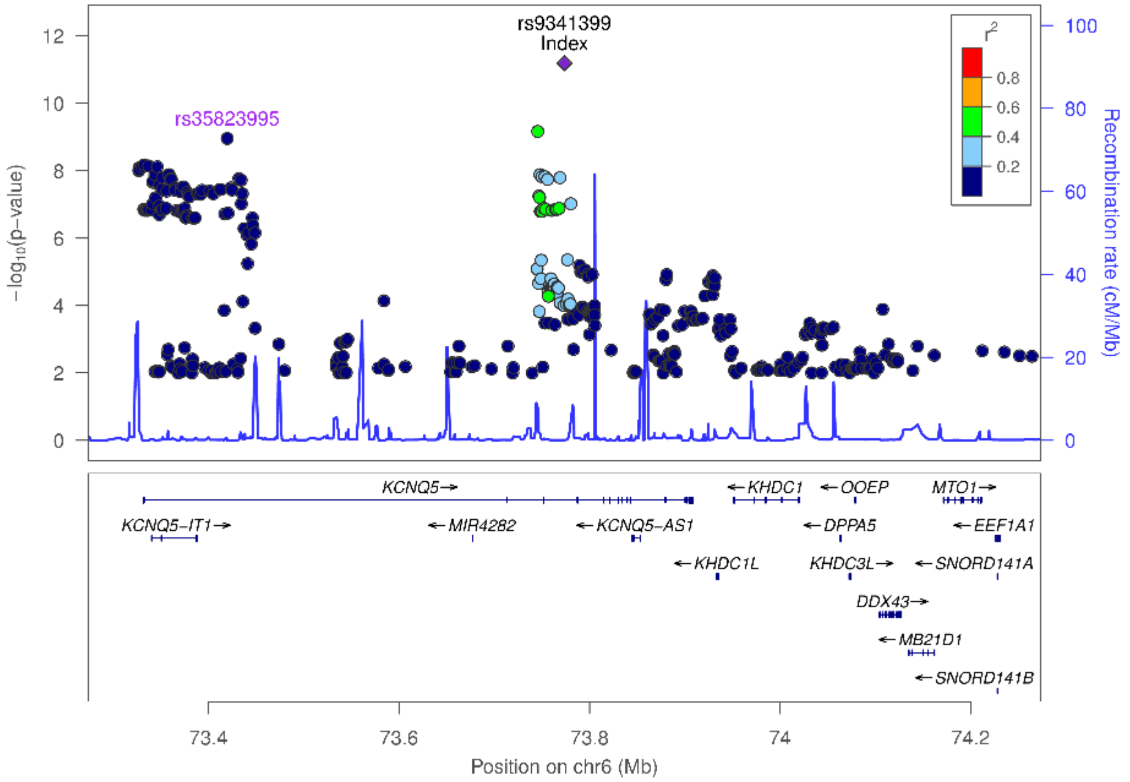
rs9341399
Index
rs35823995
r2
0.8
0.6
0.4
0.2
12
10
8
6
4
2
0
−log10(p−value)
100
80
60
40
20
0
Recombination rate (cM/Mb)
KCNQ5
KCNQ5-IT1
MIR4282
KCNQ5-AS1
KHDC1L
KHDC1
OOEP
DPPA5
KHDC3L
DDX43
MB21D1
MTO1
EEF1A1
SNORD141A
SNORD141B
73.4
73.6
73.8
74
74.2
Position on chr6 (Mb)

xxxiii)

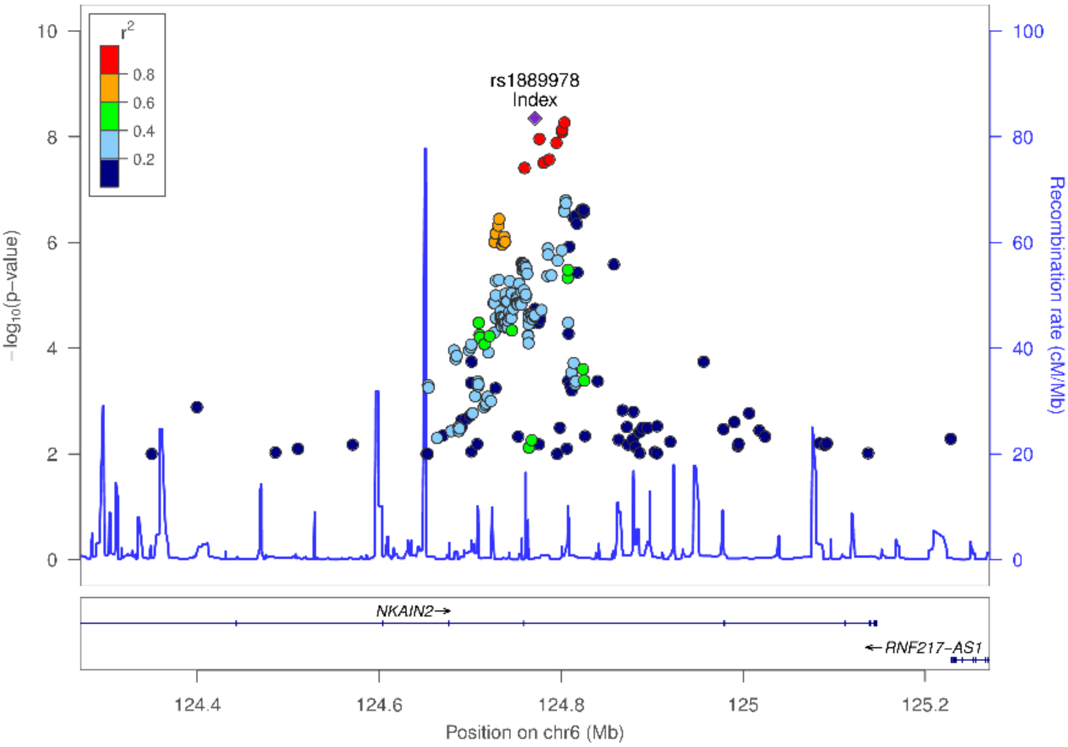

xxxiv)

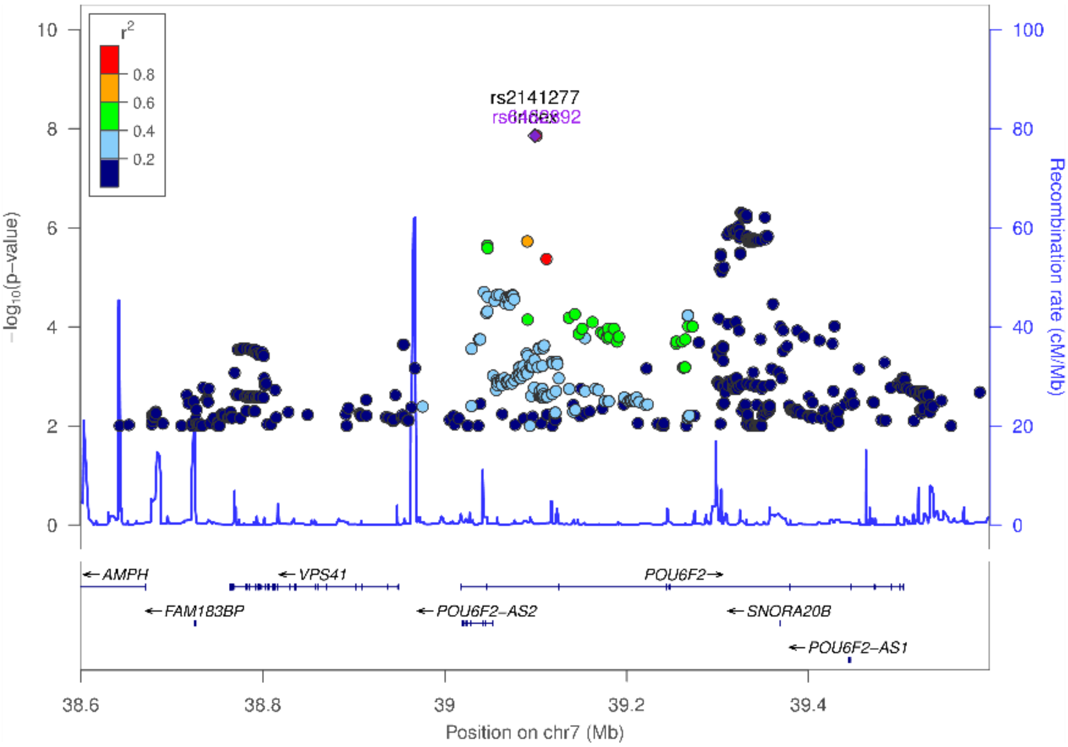

xxxv)

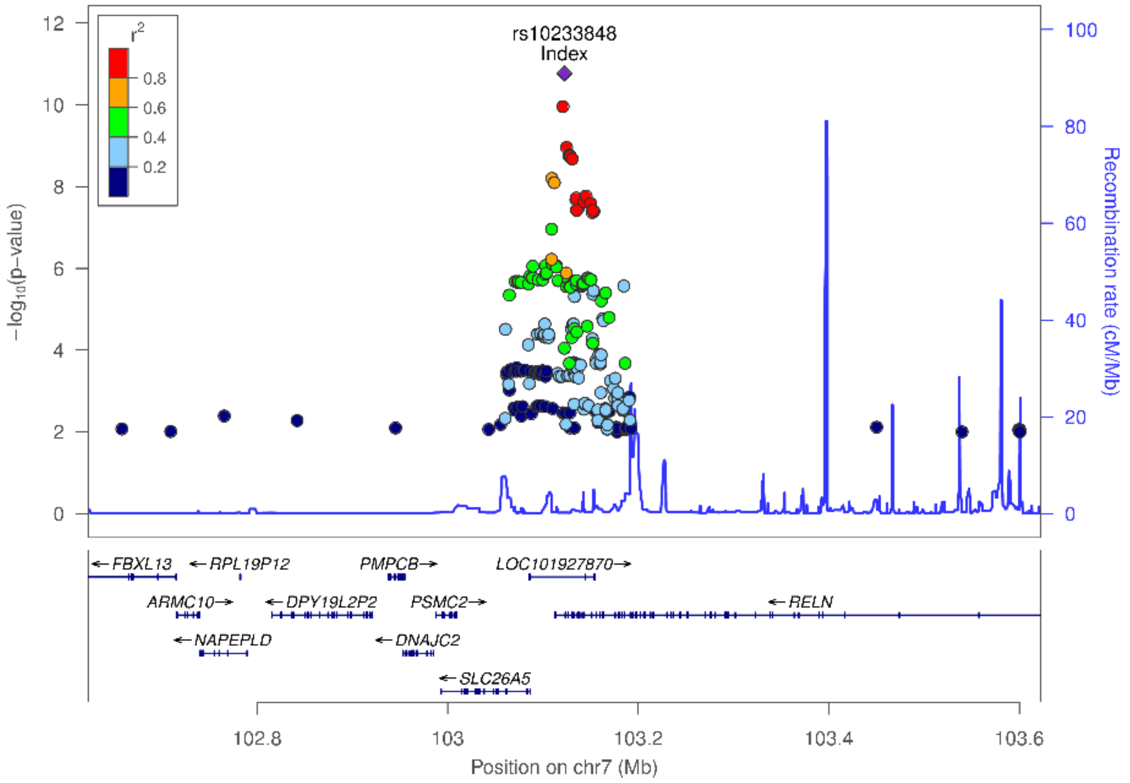

rs10233848
Index
r²
0.8
0.6
0.4
0.2
−log10(p−value)
Recombination rate (cM/Mb)
FBXL13
RPL19P12
PMPCB
LOC101927870
ARMC10
DPY19L2P2
PSMC2
RELN
NAPEPLD
DNAJC2
SLC26A5
102.8
103
103.2
103.4
103.6
Position on chr7 (Mb)

xxxvi)

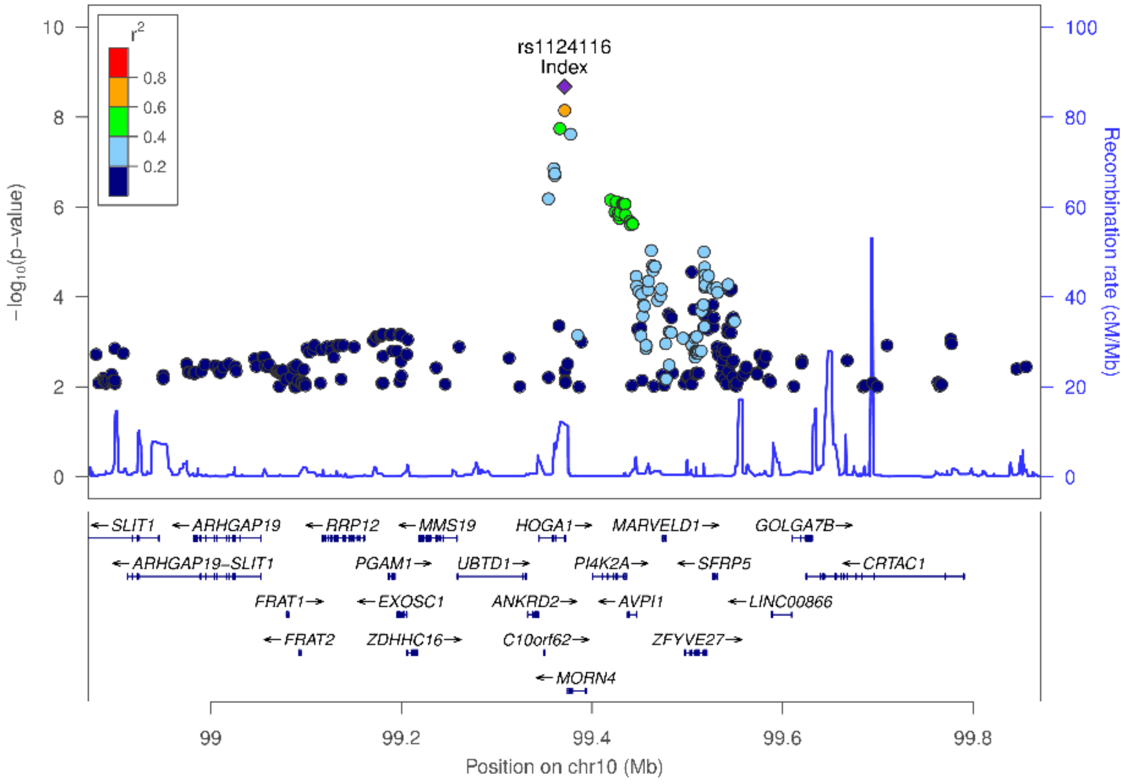

rs1124116
Index
r²
0.8
0.6
0.4
0.2
−log10(p−value)
Recombination rate (cM/Mb)
SLIT1
ARHGAP19
RRP12
MMS19
HOGA1
MARVELD1
GOLGA7B
ARHGAP19−SLIT1
PGAM1
UBTD1
PI4K2A
SFRP5
CRTAC1
FRAT1
EXOSC1
ANKRD2
AVPI1
LINC00866
FRAT2
ZDHHC16
C10orf62
ZFYVE27
MORN4
99
99.2
99.4
99.6
99.8
Position on chr10 (Mb)

xxxvii)

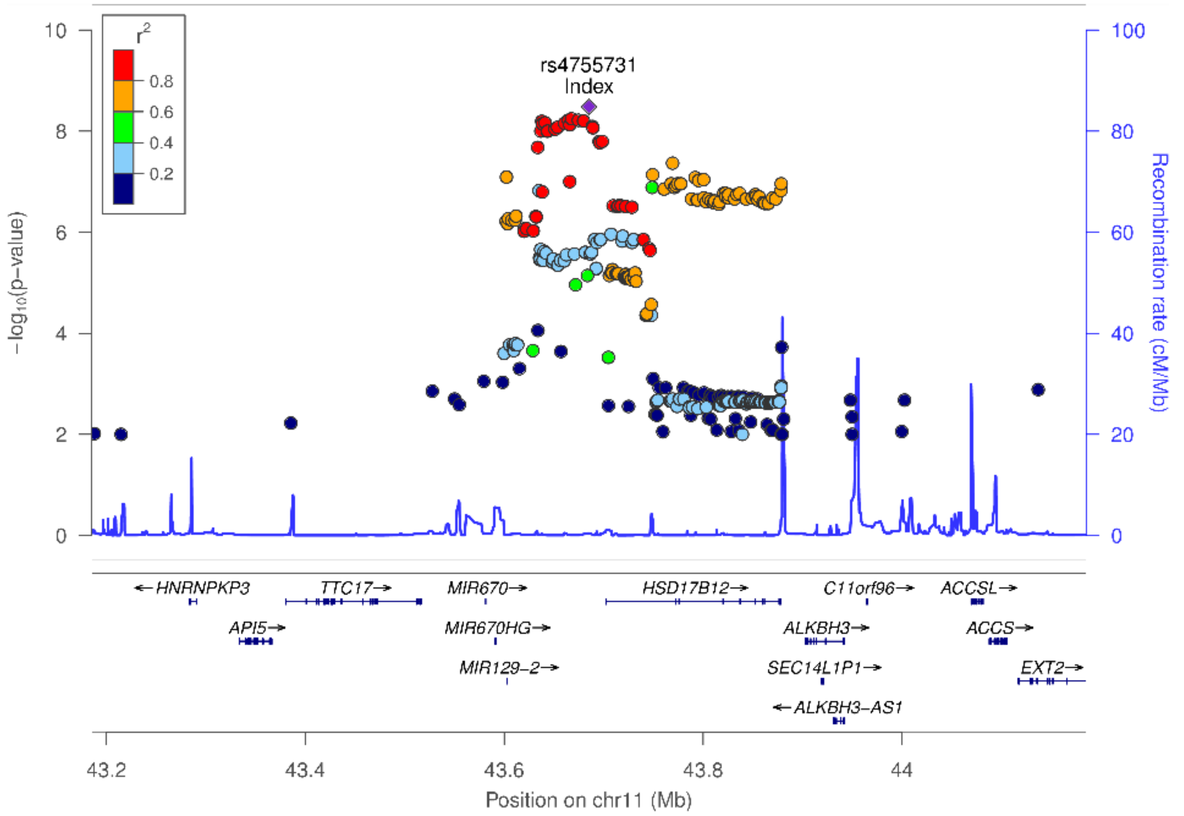

xxxviii)

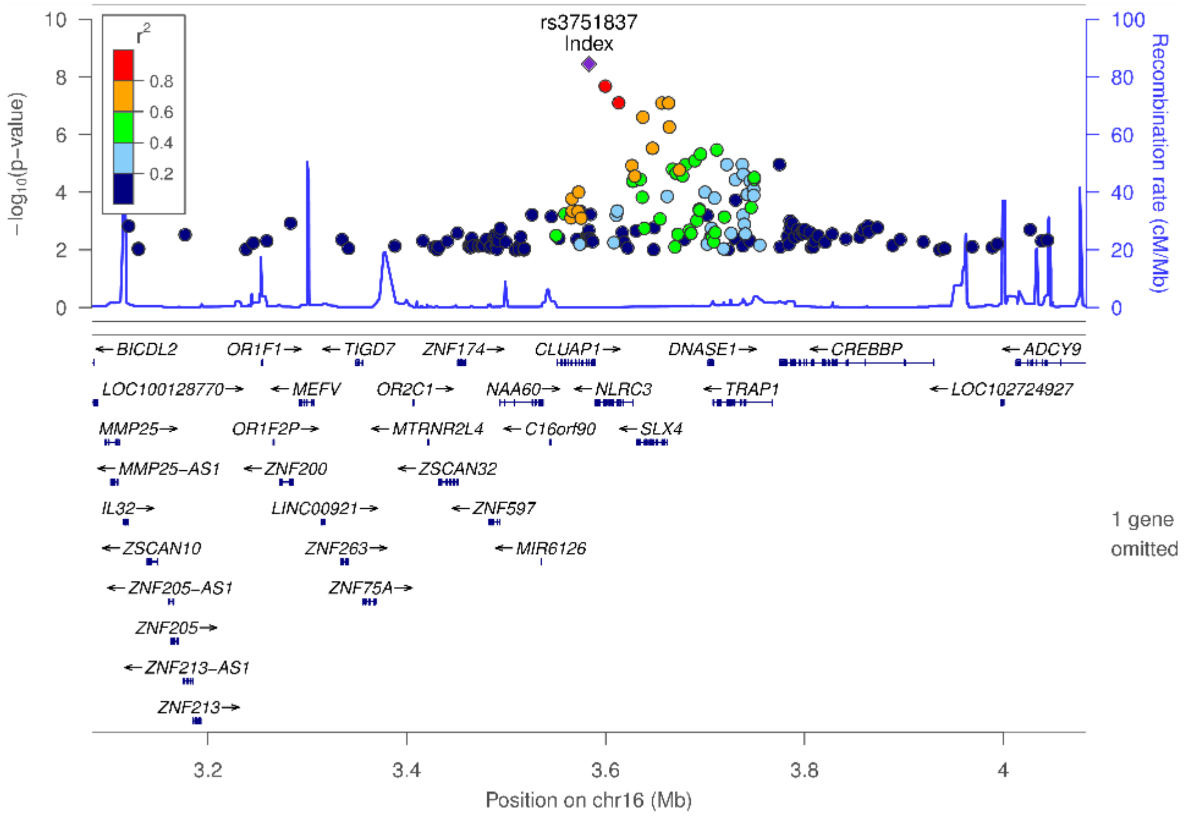

xxxix)

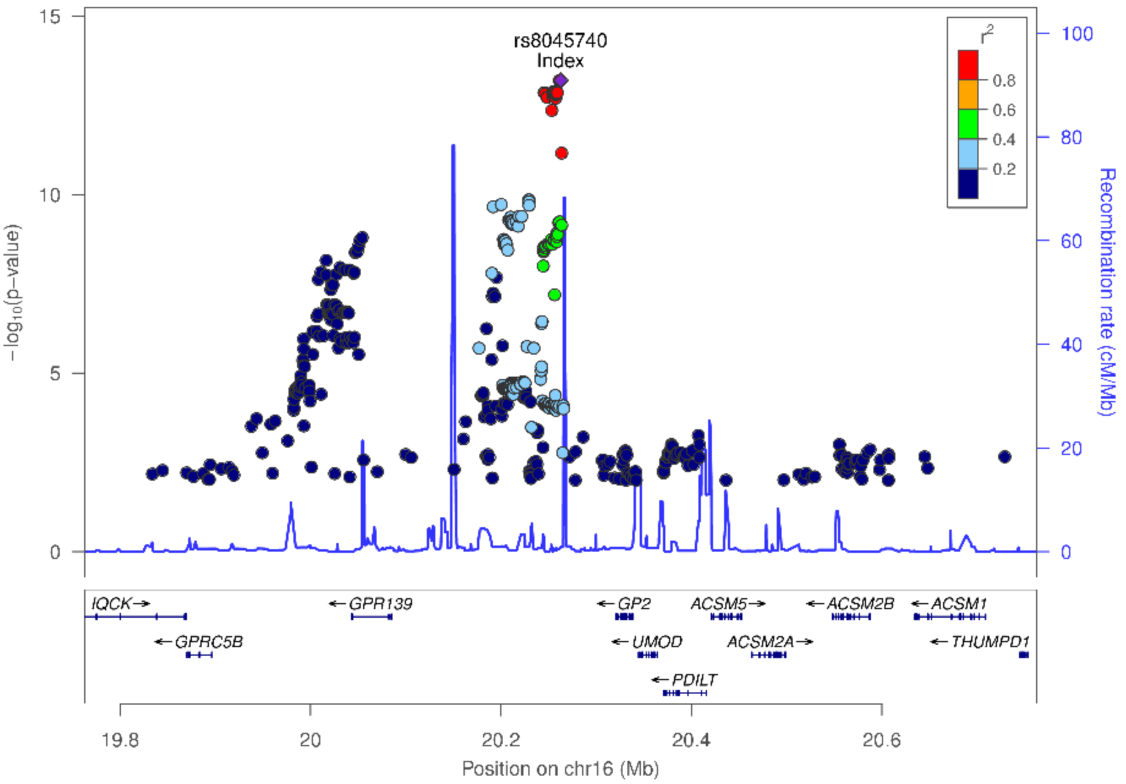
rs8045740
Index
r2
0.8
0.6
0.4
0.2
−log10(p−value)
Recombination rate (cM/Mb)
IQCK
GPRC5B
GPR139
GP2
UMOD
PDILT
ACSM5
ACSM2A
ACSM2B
ACSM1
THUMPD1
Position on chr16 (Mb)

xl)

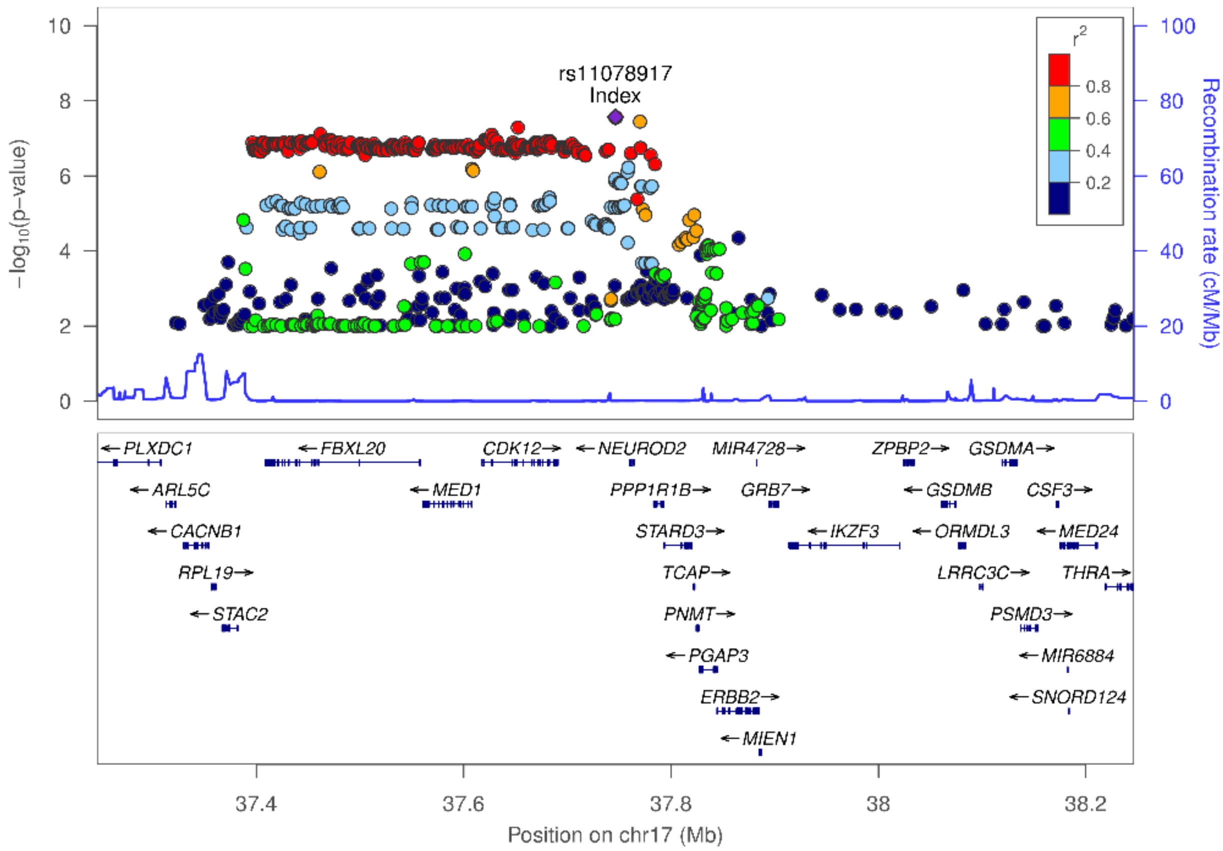
rs11078917
Index
r2
0.8
0.6
0.4
0.2
−log10(p−value)
Recombination rate (cM/Mb)
PLXDC1
ARL5C
CACNB1
RPL19
STAC2
FBXL20
MED1
CDK12
NEUROD2
PPP1R1B
STARD3
TCAP
PNMT
PGAP3
ERBB2
MIEN1
MIR4728
GRB7
IKZF3
ZPBP2
GSDMB
ORMDL3
LRRC3C
GSDMA
CSF3
MED24
THRA
PSMD3
MIR6884
SNORD124
Position on chr17 (Mb)

xli)

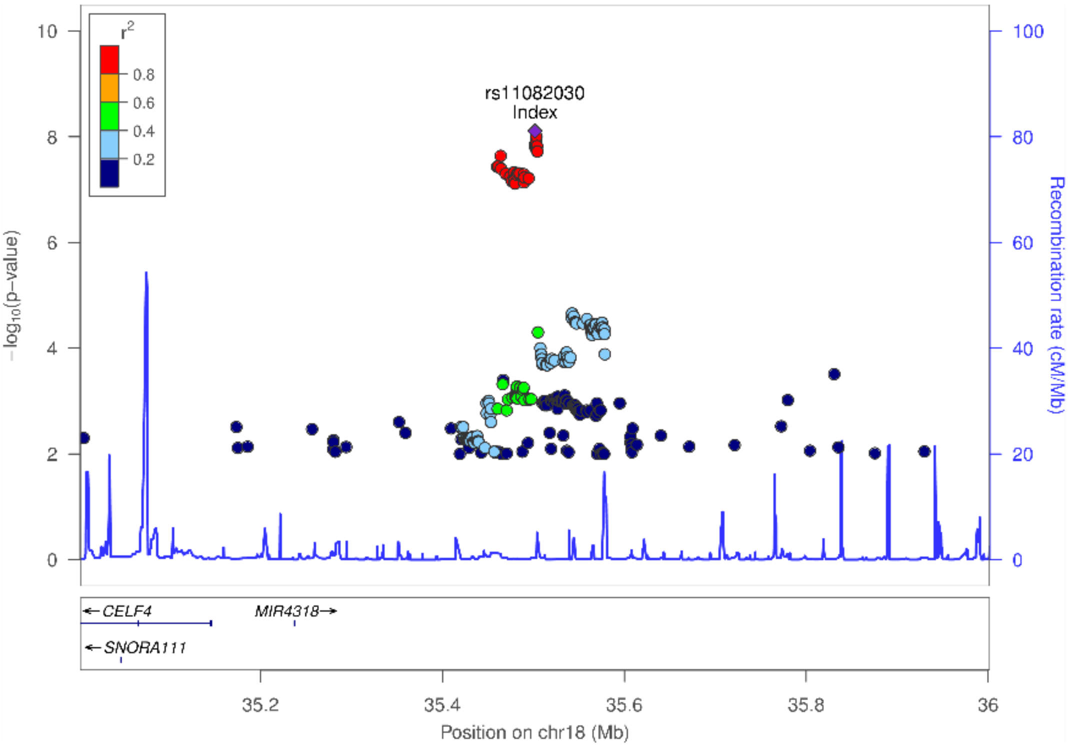

r2
0.8
0.6
0.4
0.2
rs11082030
Index
−log10(p−value)
Recombination rate (cM/Mb)
CELF4
MIR4318
SNORA111
35.2
35.4
35.6
35.8
36
Position on chr18 (Mb)

xlii)

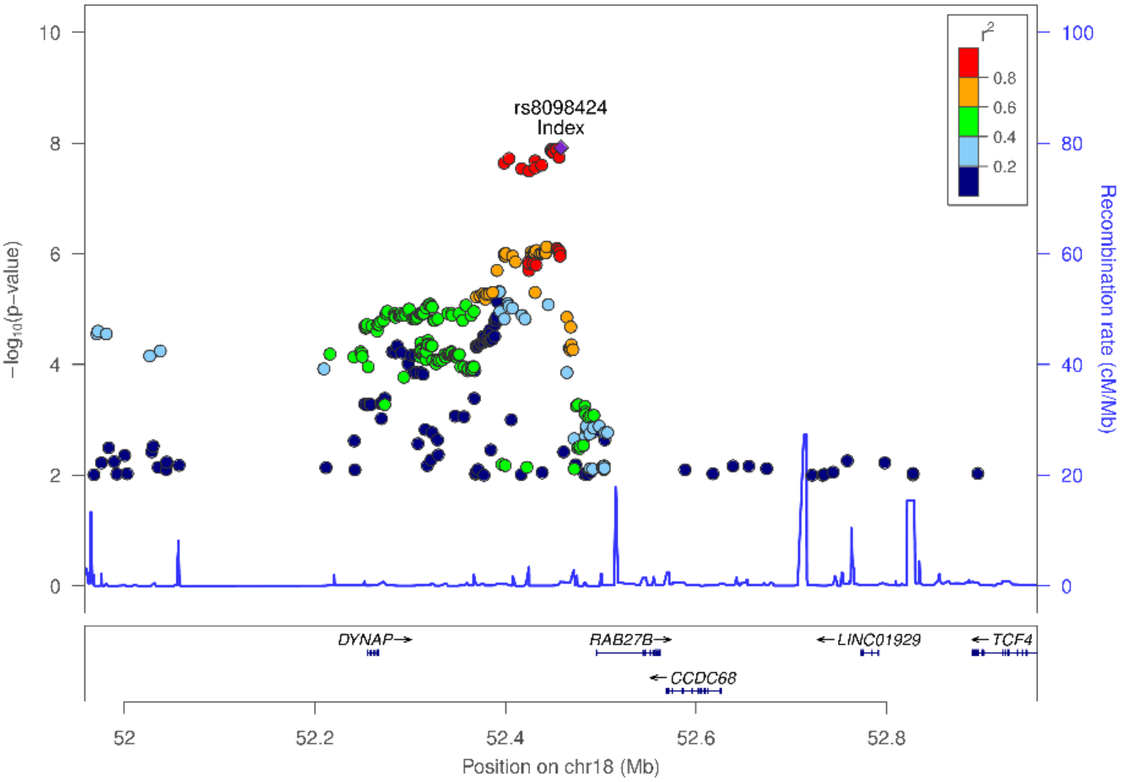

r2
0.8
0.6
0.4
0.2
rs8098424
Index
−log10(p−value)
Recombination rate (cM/Mb)
DYNAP
RAB27B
CCDC68
LINC01929
TCF4
52
52.2
52.4
52.6
52.8
Position on chr18 (Mb)

xliii)

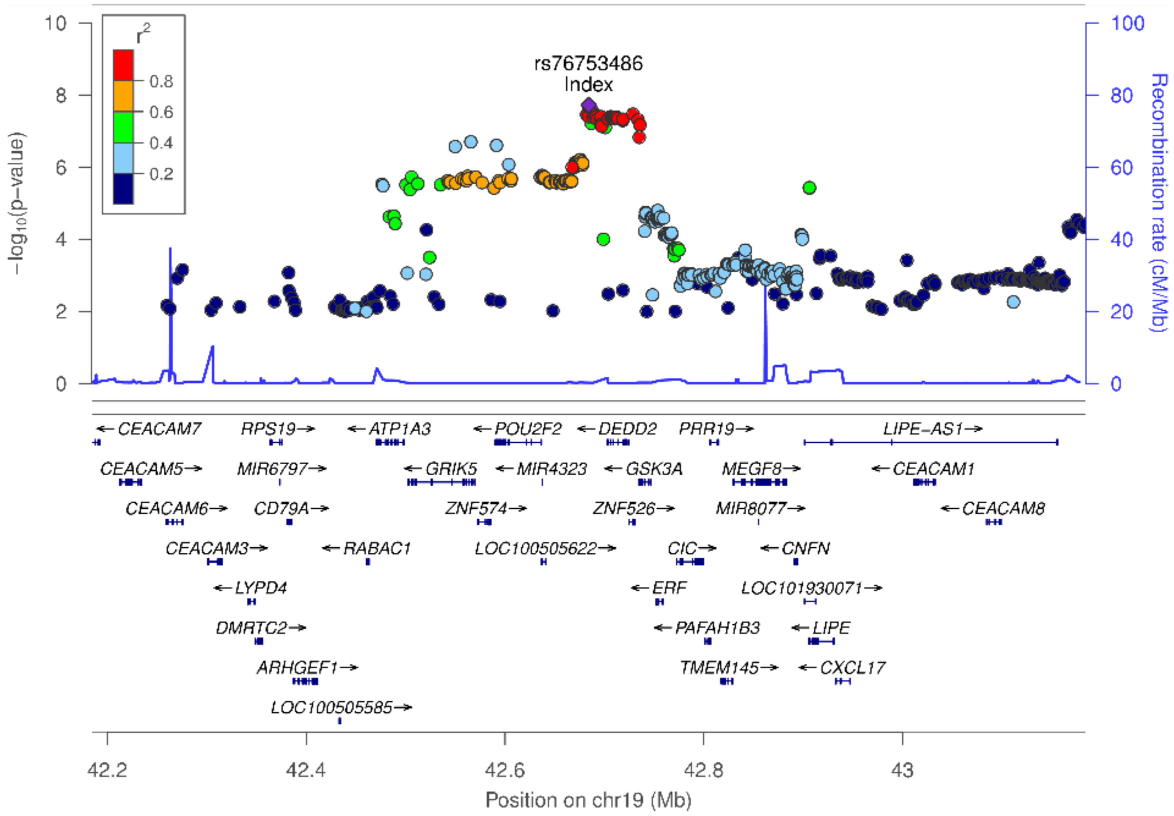
rs76753486
Index
r2
0.8
0.6
0.4
0.2
−log10(p−value)
Recombination rate (cM/Mb)
CEACAM7
RPS19
ATP1A3
POU2F2
DEDD2
PRR19
LIPE−AS1
CEACAM5
MIR6797
GRIK5
MIR4323
GSK3A
MEGF8
CEACAM1
CEACAM6
CD79A
ZNF574
ZNF526
MIR8077
CEACAM8
CEACAM3
RABAC1
LOC100505622
CIC
CNFN
LYPD4
ERF
LOC101930071
DMRTC2
PAFAH1B3
LIPE
ARHGEF1
TMEM145
CXCL17
LOC100505585
42.2
42.4
42.6
42.8
43
Position on chr19 (Mb)

xliv)

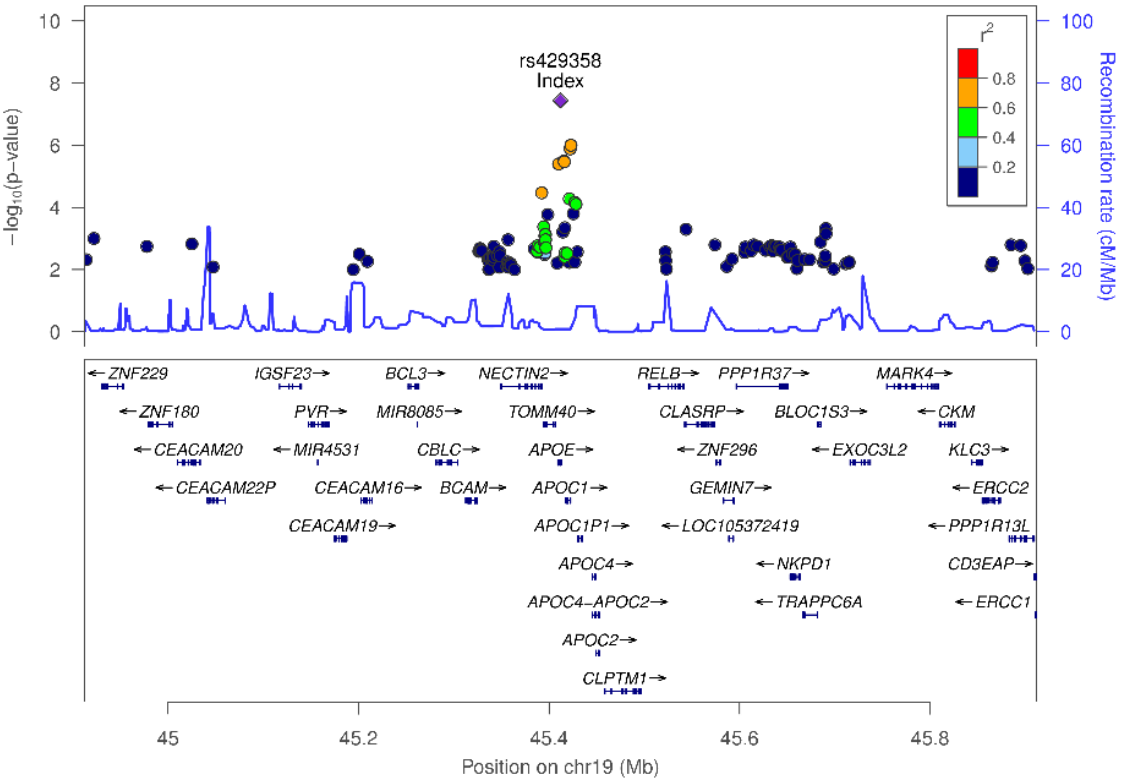
rs429358
Index
r2
0.8
0.6
0.4
0.2
−log10(p−value)
Recombination rate (cM/Mb)
ZNF229
IGSF23
BCL3
NECTIN2
RELB
PPP1R37
MARK4
ZNF180
PVR
MIR8085
TOMM40
CLASRP
BLOC1S3
CKM
CEACAM20
MIR4531
CBLC
APOE
ZNF296
EXOC3L2
KLC3
CEACAM22P
CEACAM16
BCAM
APOC1
GEMIN7
ERCC2
CEACAM19
APOC1P1
LOC105372419
PPP1R13L
APOC4
NKPD1
CD3EAP
APOC4−APOC2
TRAPPC6A
ERCC1
APOC2
CLPTM1
45
45.2
45.4
45.6
45.8
Position on chr19 (Mb)

xlv)

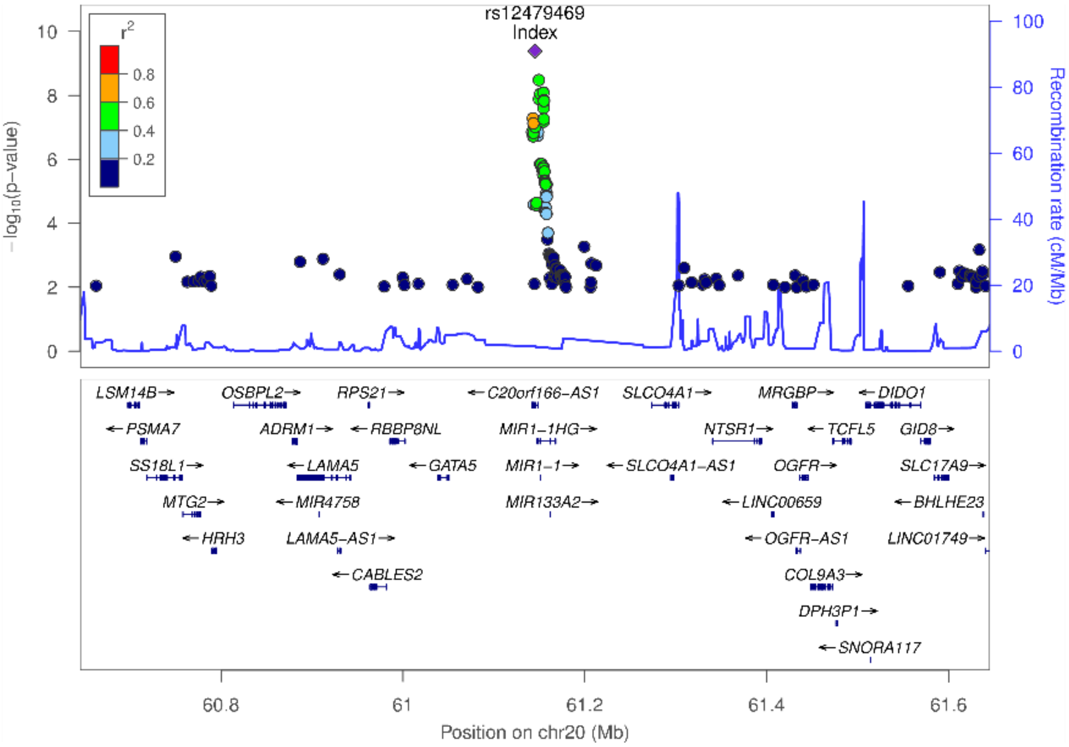
rs12479469
Index
r2
0.8
0.6
0.4
0.2
−log10(p−value)
Recombination rate (cM/Mb)
LSM14B→
OSBPL2→
RPS21→
←C20orf166−AS1
SLCO4A1→
MRGBP→
←DIDO1
←PSMA7
ADRM1→
←RBBP8NL
MIR1−1HG→
NTSR1→
←TCFL5
GID8→
SS18L1→
←LAMA5
←GATA5
MIR1−1→
←SLCO4A1−AS1
OGFR→
SLC17A9→
MTG2→
←MIR4758
MIR133A2→
←LINC00659
←BHLHE23
←HRH3
LAMA5−AS1→
←OGFR−AS1
LINC01749→
←CABLES2
COL9A3→
DPH3P1→
←SNORA117
60.8
61
61.2
61.4
61.6
Position on chr20 (Mb)

xlvi)

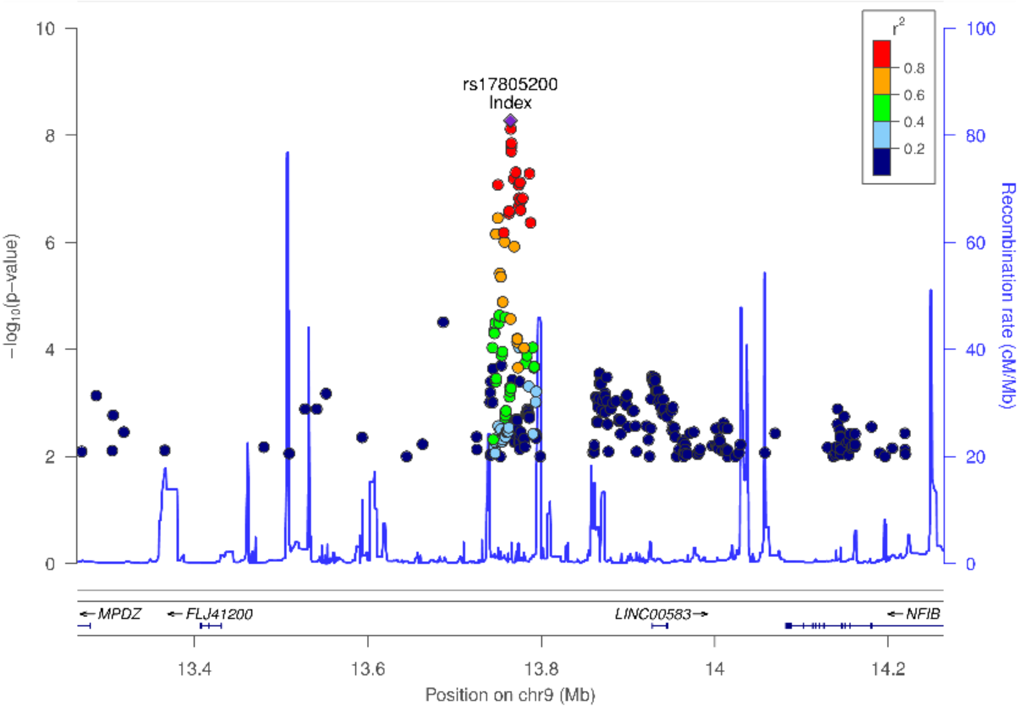
rs17805200
Index
r2
0.8
0.6
0.4
0.2
−log10(p−value)
Recombination rate (cM/Mb)
←MPDZ
←FLJ41200
LINC00583→
←NFIB
13.4
13.6
13.8
14
14.2
Position on chr9 (Mb)

xlvii)

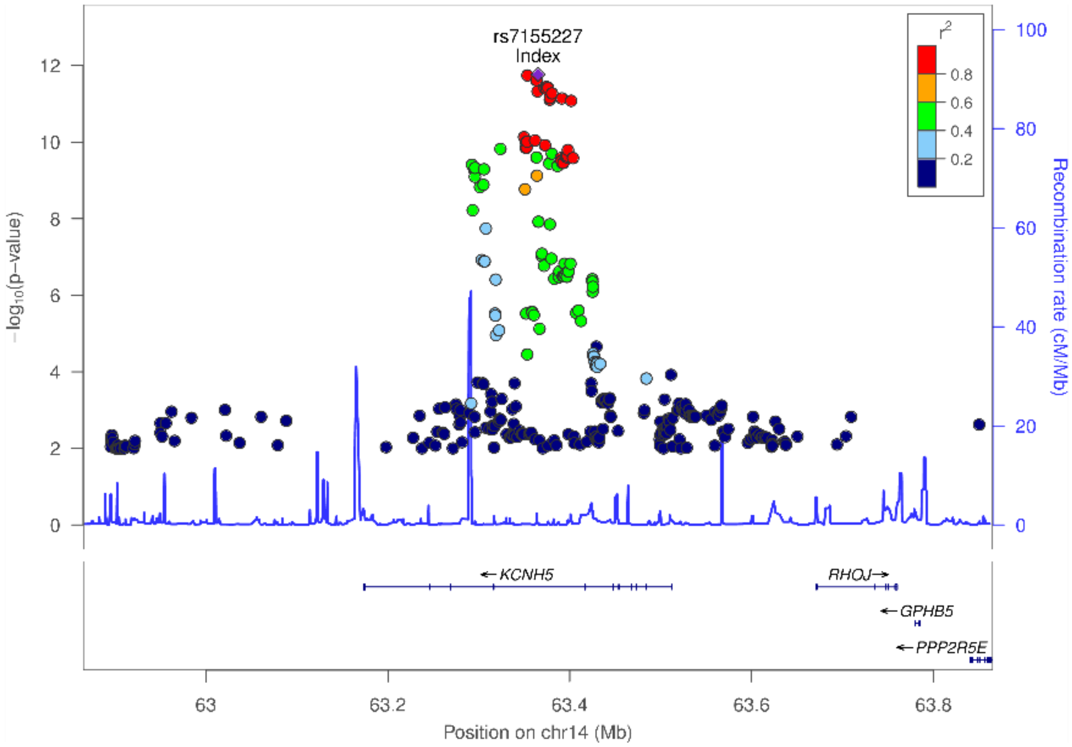

Supplementary Figure 2. Locus zoom plots for associated loci across all traits. Plots show the i) *RGS16/RNASEL*, ii) *MEIS1*, iii) *C1D*, iv) *BTBD9*, v) *CPNE8/ALG10B* and vi) *TOX3* loci for L5 timing, vii) *DPYD*, viii) *MEIS1*, ix) *PAX8*, x) *PDE11A*, xi) *LOC101928519*, xii) *BTBD9*, xiii) *MSRA*, xiv) *ANK1*, xv) *LRP12/ZFPM2*, xvi) *CAND1* and xvii) *KCNH5* loci for sleep duration, xviii) *CAB39* locus for sleep midpoint, xix) *MEIS1*, xx) *PAX8*, xxi) *PDE11A*, xxii) *PHF7* and xxiii) *FOXL2* loci for sleep efficiency, xxiv) *CPNE8/ALG10B* locus for M10 timing, xxv) *ACP1/SH3YL1*, xxvi) *SUMF1/SETMAR*, xxvii) *HTR1F*, xxviii) *IL20RB/SOX14*, xxix) *RNF4*, xxx) *BANK1*, xxxi) *HTR1A/RNF180*, xxxii) *KCNQ5*, xxxiii) *NKAIN2*, xxxiv) *POU6F2*, xxxv) *RELN*, xxxvi) *HOGA1/MORN4*, xxxvii) *HSD17B12*, xxxviii) *CLUAP1*, xxxix) *GPR139*, xl) *NEUROD2*, xli) *CELF4*, xlii) *RAB27B*, xliii) *DEDD2/ZNF526*, xliv) *APOE* and xlv) *MIR133A2* loci for number of sleep episodes, and the xlvi) *MPDZ/NFIB* and xlvii) *KCNH5* loci for diurnal inactivity. Index (most significant) variants are labelled in black text and credible causal variants identified by FINEMAP (if any) are labelled in purple text. Variants with P>0.01 were excluded from the plots for clarity.

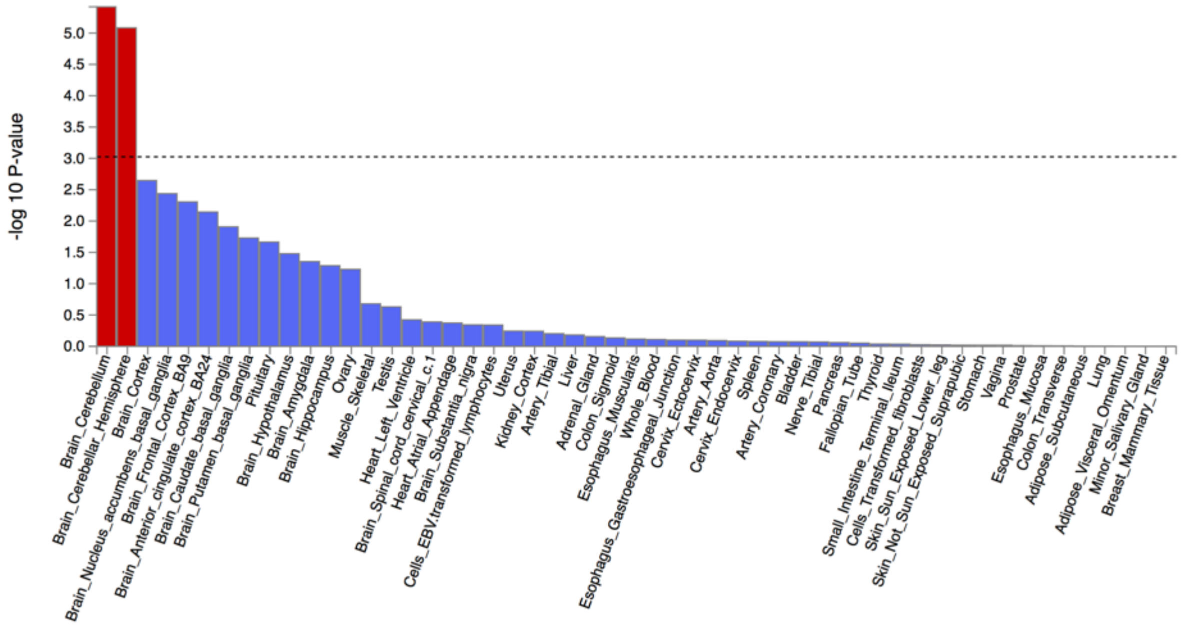

Supplementary Figure 3. Tissue enrichment of genes at associated loci for accelerometer-based sleep duration. Genes were identified using MAGMA and then analysed for enrichment using the GTEx RNA-seq database (v6) to investigate expression across 53 tissue types.

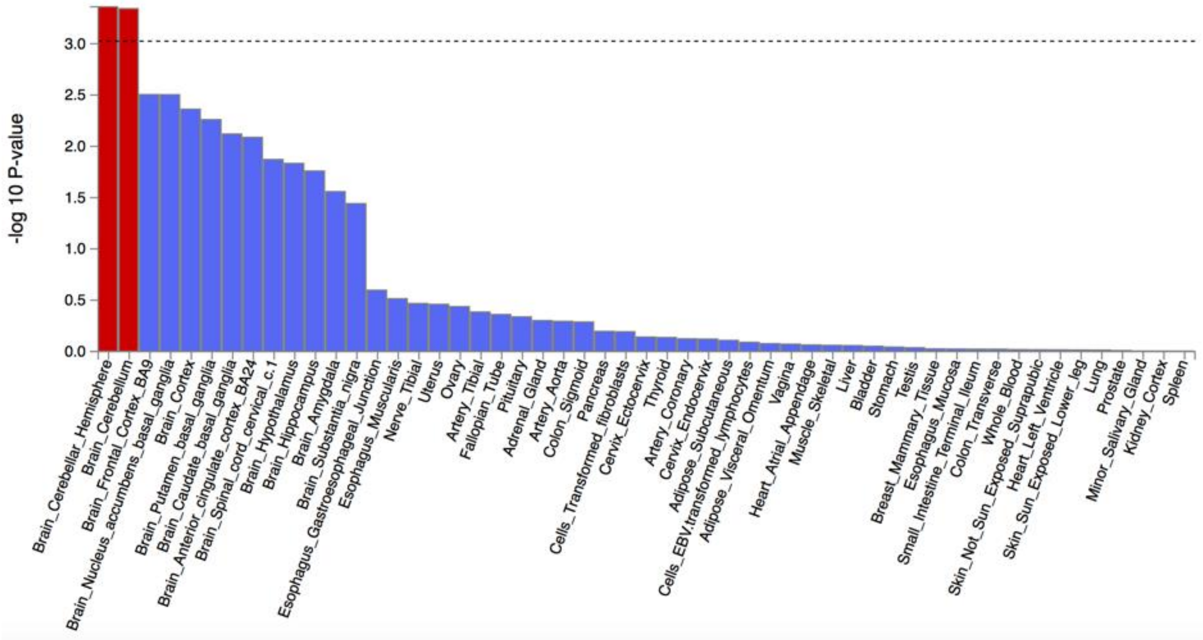

Supplementary Figure 4. Tissue enrichment of genes at associated loci for the number of sleep episodes. Genes were identified using MAGMA and then analysed for enrichment using the GTEx RNA-seq database (v6) to investigate expression across 53 tissue types.
