# Supplementary material for: Genetic studies of accelerometer-based sleep measures yield new insights into human sleep behaviour
Source: Nat Commun. 2019 Apr 5;10:1585. doi: 10.1038/s41467-019-09576-1 (PMC6451011; doi:10.1038/s41467-019-09576-1)
Supplement: Supplementary file 3 — Description of Additional Supplementary Files [file 41467_2019_9576_MOESM3_ESM.pdf]

Description of Additional Supplementary Files

Supplementary Data 1. Association statistics for the 47 signals discovered in UKB Biobank in the Whitehall II and CoLaus and Rotterdam I, II, and III replication cohorts. Meta-analysis of the results across the studies are also provided. Grey cells indicate data unavailable from the respective study.

Supplementary Data 2. Analysis of the combined genetic effects from Supplementary Data 1 within the Whitehall II, COLAUS and Rotterdam replication studies.

Supplementary Data 3. Results from sensitivity analyses performed for the 47 signals reaching P<5x10-8 in the UK Biobank. All analyses were performed in a set of unrelated European where individuals from related pairs were removed at random. Association tests were carried out for all phenotypes on both the raw scale and inverse-normalised scale. Sensitivity analysis included: 1) in males only, 2) in females only, 3) in those lower than median age at actigraphy (63.7 years), 4) in those greater than or equal to the median age, 5) in all European unrelated but adjusting for BMI in addition to standard adjustments, 6) in all European unrelated but also adjusting for BMI and lifestyle factors, and 7) excluding those reporting shift work, having self-report or hospital-recorded mental health or sleep disorders, and those taking anxiolytic, antipsychotic, antidepressant or sleep medication. Lifestyle adjustments for analysis (6) and exclusions for analysis (7) are described in greater detail in the Supplementary Methods.

Supplementary Data 4. Association result cross-tabulation against other traits for the 47 SNPs representing genetic associations reaching P<5x10-8 in UK Biobank. Cross-tabulation also includes results based on the latest self-report chronotype meta-analyses (Jones et al., Nat. Commun. 2019; doi.org/10.1038/s41467-018-08259-7), self-report Insomnia GWAS (Lane et al., Nature Genet. 2019; doi.org/10.1038/s41588-019-0361-7) and sleep duration GWAS (Dashti et al., Nat. Commun. 2019; doi.org/10.1038/s41467-019-08917-4) in UK Biobank are also provided.

Supplementary Data 5. Fine-mapped loci with at least one plausible variant (log10 Bayes' Factor > 2) with variant annotations from GTEx and Alamut. For the GTEx annotations, a gene was listed if there was significant evidence of enrichment of expression in a) at least one brain tissue or b) the meta-analysis of expression across all tissues. In the brain eQTL analysis, we report genes for which there is significant evidence of association with our fine-mapped variant and expression levels of the gene in one or more brain tissues. To report the lead eQTL variant r2 with our fine-mapped variant, we first identify the tissue for which our variant has the strongest evidence (smallest P-value) of effect on expression and then report the r2 between our variant and the variant most strongly associated with expression levels in that tissue. For the all-tissue meta-analysis, we report the gene if there is significant evidence of association in the meta-analysis across all tissues, reporting the r2 between our fine-mapped variant and the one with the strongest evidence of effect on expression across all tissues.

Supplementary Data 6. Results from Mendelian Randomization (MR) analyses of Restless Legs Syndrome exposure against multiple outcomes using 4 methods: 1) using Inverse-variance (IV) weighted MR, 2) Egger MR, 3) Weighted Median (WM) MR and 4) Penalised-weighted mean (PWM).

Supplementary Data 7. Genetic correlation results for the 8 accelerometer-derived sleep traits against 234 LD Hub phenotypes, ordered by P-value. P-values reaching Bonferroni significance (P<0.05/(8*234)) in bold.

Supplementary Data 8. Mendelian Randomization analyses testing causality of seven genetically correlated traits on accelerometer-based sleep outcomes.

Supplementary Data 9. Mendelian Randomization analyses testing causality of four accelerometer-based sleep exposures on genetically correlated traits. Sleep exposures with <3 genome-wide associations at P<5x10-8 or outcomes with <3 genetic instruments available in published datasets (highlighted grey) were excluded from this analysis.

Supplementary Data 10. R wrapper script used to generate the sleep and activity phenotypes described in the manuscript "Genetic studies of accelerometer-based sleep measures yield new insights into human sleep behaviour".
